# D/PVA/I-1 as an Antibiotic Adjuvant: In Vitro Synergy and Membrane Permeabilization in MDR Bacteria

**DOI:** 10.3390/ph19081300

**Published:** 2026-08-17

**Authors:** Ardak Jumagaziyeva, Seitzhan Turganbay, Anar Seisembekova, Daniil Shepilov, Zhanar Iskakbayeva, Sabina Kenesheva, Saltanat Jumabayeva, Gaukhar Askhatkyzy, Nurdaulet Temir, Abdurashit Khamidulin, Alexandr Ilin

**Affiliations:** 1 Scientific Center for Anti-Infectious Drugs JSC, Almaty 050060, Kazakhstan; seysembekovaanar@gmail.com (A.S.); shepilov2002@gmail.com (D.S.); zhanara_07_74@mail.ru (Z.I.); silentium_n@bk.ru (S.K.); salta_albann@mail.ru (S.J.); askhatkyzyg@mail.ru (G.A.); btnurdaulet@gmail.com (N.T.); khamidulin05@gmail.com (A.K.); ilin_ai@mail.ru (A.I.); 2 Faculty of Natural Sciences, Kazakh National Women’s Teacher Training University, Almaty 050000, Kazakhstan; 3 “One Belt, One Road” Petroleum Engineering, Kazakh-British Technical University, Almaty 050000, Kazakhstan; 4 Department of Fundamental Medicine, Farabi University, Almaty 050000, Kazakhstan

**Keywords:** antimicrobial resistance, multidrug-resistant bacteria, MDR, antibiotic potentiation, synergism, FICI, time-kill assay, D/PVA/I-1, membrane permeability, ESKAPE pathogens

## Abstract

**Background:** Antimicrobial resistance (AMR) is among the most pressing challenges in modern infectious medicine, driving progressive failure of standard antibacterial therapy and rising mortality from infections caused by multidrug-resistant (MDR) pathogens. Antibiotic potentiation through adjuvant compounds capable of restoring the activity of existing drugs represents a promising strategy to overcome resistance without developing fundamentally new antibacterial molecules. This study aimed to evaluate the antibiotic-potentiating activity of a dextrin/polyvinyl alcohol/iodine complex (D/PVA/I-1), developed at the Scientific Center for Anti-Infectious Drugs JSC (Almaty, Kazakhstan), against clinically relevant MDR reference strains. **Methods:** Nine reference strains, *Staphylococcus aureus* (ATCC 33591, BAA-39), *Escherichia coli* (ATCC BAA-196, BAA-2523), *Klebsiella pneumoniae* (ATCC BAA-2524, 700603), *Acinetobacter baumannii* (ATCC BAA-1790), *Streptococcus pneumoniae* (ATCC BAA-660), and *Haemophilus influenzae* (ATCC 33930), and two clinical isolates, *P. aeruginosa* SCAID PHRX1-2019 and *E. coli* SCAID WND1-2021, were tested. Antibiotic-potentiating activity was assessed by checkerboard assay with calculation of the fractional inhibitory concentration index (FICI); bactericidal kinetics were evaluated by time-kill analysis. The effect of D/PVA/I-1 on cytoplasmic membrane permeability was investigated using a crystal violet uptake assay. **Results:** Of 45 D/PVA/I-1–antibiotic combinations tested across nine antibiotics, synergy (FICI ≤ 0.5) was demonstrated in 44.4% of cases, partial synergy in 48.9%, and additive effects in 6.7%; no antagonistic interactions were detected. The most pronounced potentiating effect occurred against Gram-positive pathogens, particularly MRSA strains (66.7% synergistic combinations). Time-kill analysis confirmed suppression of the regrowth phenotype characteristic of MDR strains under monotherapy and restoration of bactericidal activity against antibiotics to which strains exhibited intrinsic resistance. D/PVA/I-1 induced a dose- and time-dependent increase in membrane permeability in both Gram-positive and Gram-negative organisms. **Conclusions:** D/PVA/I-1 is an effective broad-spectrum antibiotic potentiator and represents a promising basis for combination therapy regimens against MDR infections.

## 1. Introduction

Antimicrobial resistance (AMR) represents one of the most pressing challenges in contemporary infectious medicine. According to the World Health Organization, in 2019, AMR was directly responsible for approximately 1.27 million deaths and contributed to 4.95 million fatalities worldwide [1]. Of particular concern is the escalating resistance among ESKAPE pathogens—*Enterococcus faecium*, *Staphylococcus aureus*, *Klebsiella pneumoniae*, *Acinetobacter baumannii*, *Pseudomonas aeruginosa*, and *Enterobacter* spp.—which collectively account for the majority of nosocomial infections, with the diminishing efficacy of existing treatment regimens continuously increasing the global burden of morbidity and mortality [2].

The conventional pathway for developing novel antibiotics is increasingly failing to meet clinical needs. The discovery of new antibacterial monotherapies is hampered by exceptionally slow timelines, prohibitive costs, and limited commercial returns, resulting in a sustained deficit of investment in this field [3]. Against this backdrop, the strategy of antibiotic potentiation through adjuvant molecules has emerged as a highly relevant alternative and economically viable approach. Combining approved antibiotics with inhibitors of resistance mechanisms enables the restoration or enhancement of classical drug activity against inherently resistant pathogens, without necessitating the development of entirely new chemical entities [4].

Three principal classes of antibiotic adjuvants are currently recognized: beta-lactamase inhibitors (BLIs), efflux pump inhibitors (EPIs), and outer membrane permeabilizers—each targeting a distinct key mechanism of acquired bacterial resistance [4]. BLIs represent the most clinically validated class: combinations such as ceftazidime-avibactam and ceftolozane-tazobactam have been incorporated into treatment guidelines for infections caused by ESBL-producing *Enterobacterales* and *P. aeruginosa*. Nevertheless, the development of next-generation BLIs remains an active area of research, as clinical strains continue to acquire multiple resistance mechanisms that circumvent the activity of already-approved combinations [5].

**Figure 1:**
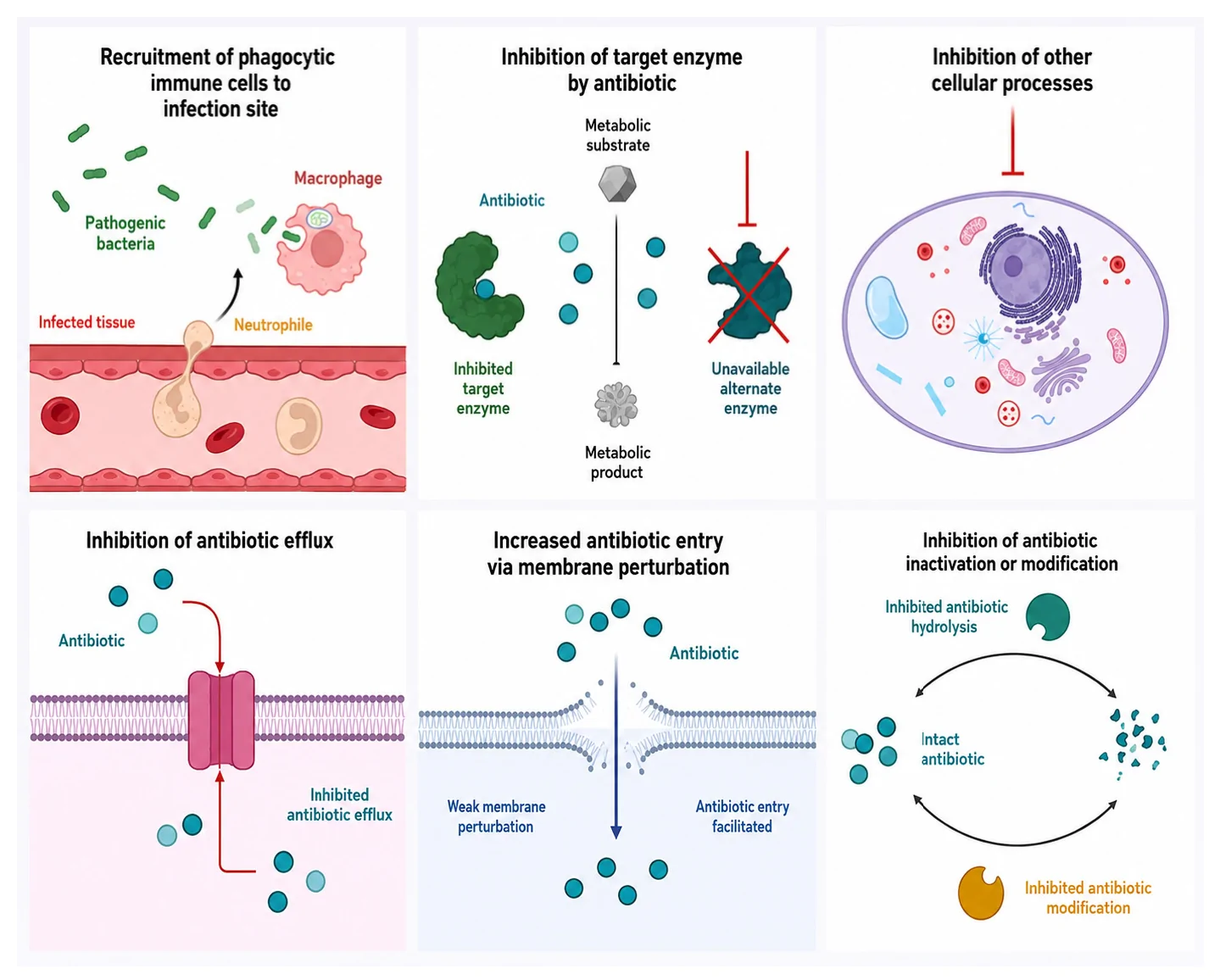
Mechanisms of potentiation by adjuvants. The figure was created using BioRender.com.

Synergistic interactions between antibiotics, or between an antibiotic and an adjuvant, offer the opportunity not only to enhance bactericidal efficacy but also to reduce dosing requirements, thereby limiting adverse effects. When carefully selected drug combinations are employed, synergistic bactericidal activity can be achieved against highly resistant strains, while such regimens are generally less costly to develop and safer to administer, given that the constituent agents have already undergone clinical evaluation [3]. Furthermore, synergistic combinations have been shown to extend the clinical lifespan of existing drugs while simultaneously reducing the risk of secondary resistance emergence [6]. Adjuvants’ mechanisms of action are presented in Figure 1.

The checkerboard assay, employing the fractional inhibitory concentration index (FICI), remains the standard method for quantitative assessment of drug interactions in vitro. The FICI is calculated as the sum of the minimal inhibitory concentrations (MICs) of each antibiotic in combination, normalized to their individual MICs, with FICI values of ≤0.5 widely accepted as the criterion for synergy according to Loewe’s additivity model [7]. Alongside the classical checkerboard approach, high-throughput screening platforms integrated with machine learning methods are increasingly being applied to accelerate the identification of synergistically active compound pairs.

Taken together, the search for effective antibiotic potentiators and the elucidation of the underlying mechanisms of drug synergy represent a highly promising avenue for addressing the AMR crisis. As previously demonstrated by our group, D/PVA/I-1 exhibited pronounced intrinsic antibacterial activity, with minimum bactericidal concentrations (MBCs) ranging from 0.33 μg/mL against Gram-positive strains (*S. aureus*, *A. baumannii*, *S. pneumoniae*) to 0.68 μg/mL against Gram-negative strains (*E. coli*, *K. pneumoniae*, *P. aeruginosa*) [8]. This antibacterial effect was attributed to the gradual release of iodine (present predominantly as triiodide ions, I_3_^−^) from the polymer matrix, which penetrates bacterial cell walls and disrupts cell wall synthesis and protein function in Gram-positive bacteria, while exerting oxidative damage to the cell membrane and DNA in Gram-negative bacteria. Regarding biocompatibility, D/PVA/I-1 was previously shown to have a favorable safety profile at lower concentrations, with moderate cytotoxicity toward the MCF7 tumor cell line and PBMCs observed only at 5 mg/mL (62.4% and 52.0% cell viability, respectively), and a pronounced cytotoxic effect on the normal MeT-5A cell line at 2.5–5.0 mg/mL (13.8–23.0% cell viability). These findings indicated that the cytotoxic threshold of D/PVA/I-1 lies well above its effective antibacterial concentrations (in the sub-μg/mL range), supporting a wide therapeutic window for topical/wound-care applications.

The aim of the present study is to evaluate the antibiotic-potentiating activity of the dextrin/polyvinyl alcohol/iodine complex (D/PVA/I-1) against clinically relevant multidrug-resistant reference strains through checkerboard assay, time-kill analysis, and cytoplasmic membrane permeability studies, with the goal of advancing our understanding of the molecular basis of potentiation and providing a foundation for the development of novel combination therapy regimens against resistant bacterial infections.

## 2. Results

### 2.1. Time-Kill Assay Results

**Figure 2:**
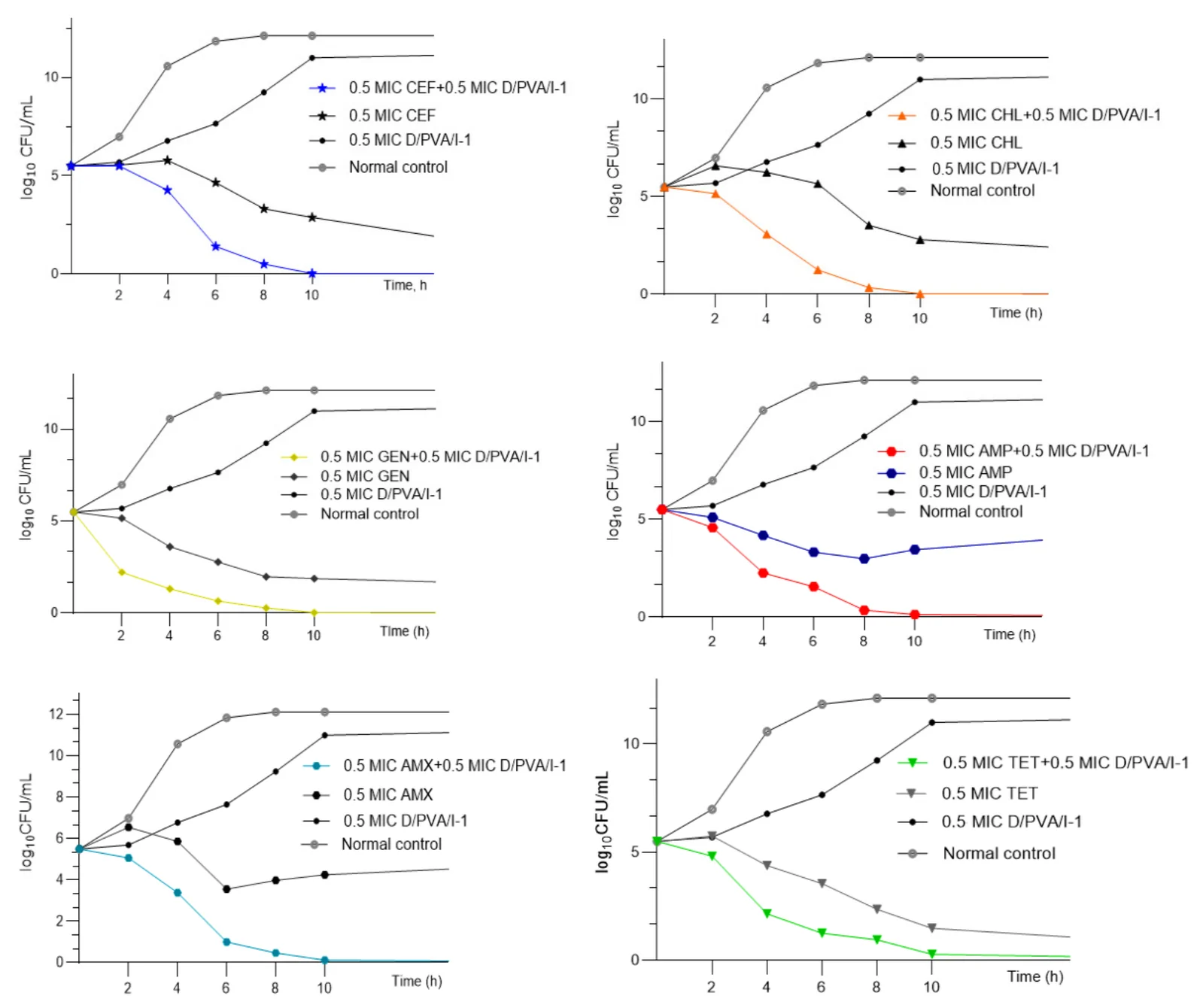
Time-kill curves for *S. aureus* ATCC BAA-39 strain in presence of D/PVA/I-1 and AB (CEF, CHL, GEN, AMP, AMX, TET) alone or in combination, and a normal control (only medium without any drug) for 24 h. The figure is original artwork created by the authors using GraphPad Prism 5 (GraphPad Software, Inc., San Diego, CA, USA).

**Figure 3:**
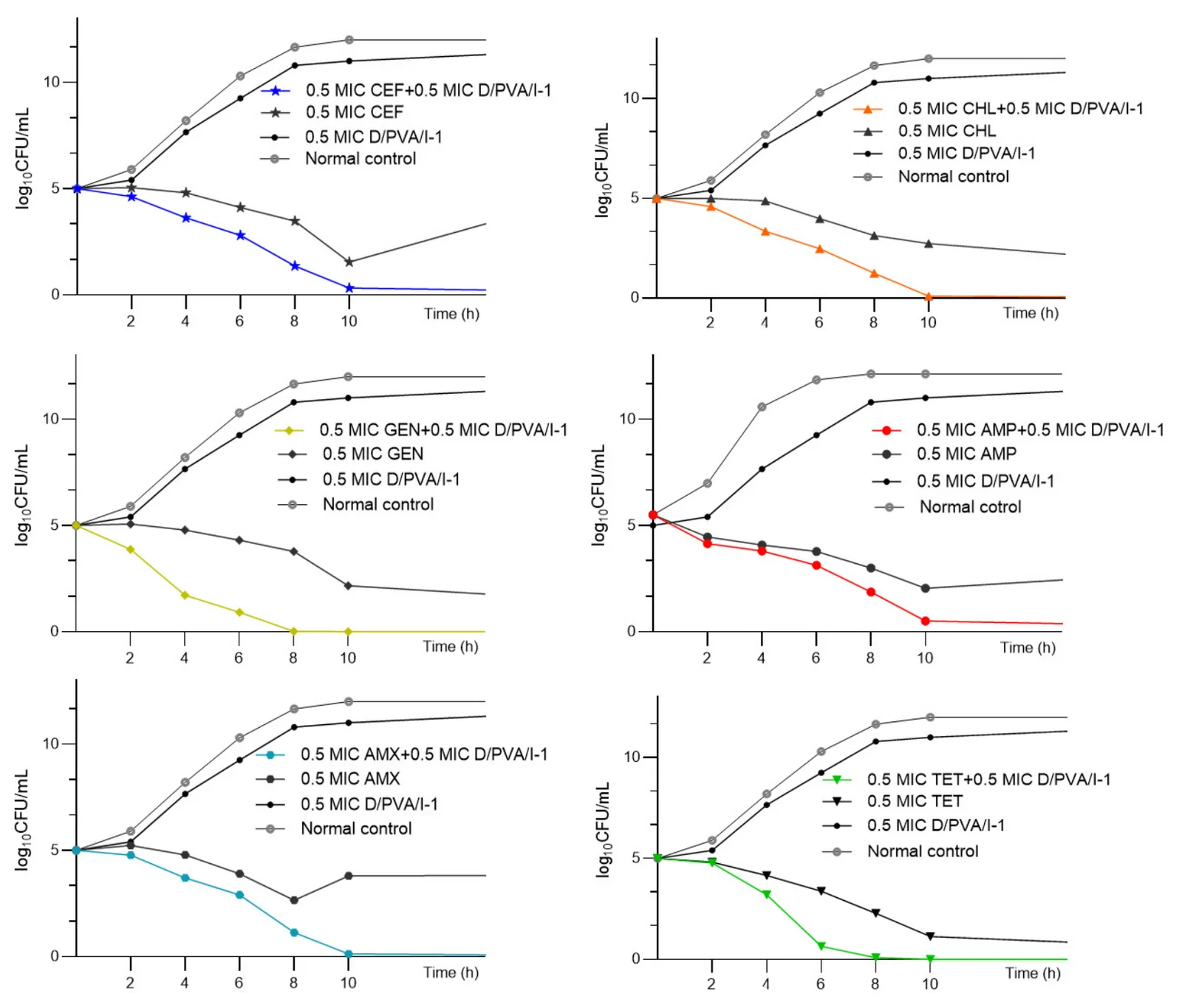
Time-kill curves for *S. aureus* ATCC 33591 strain in presence of D/PVA/I-1 and AB (CEF, CHL, GEN, AMP, AMX, TET) alone or in combination, and a normal control (only medium without any drug) for 24 h. The figure is original artwork created by the authors using GraphPad Prism 5 (GraphPad Software, Inc.).

**Figure 4:**
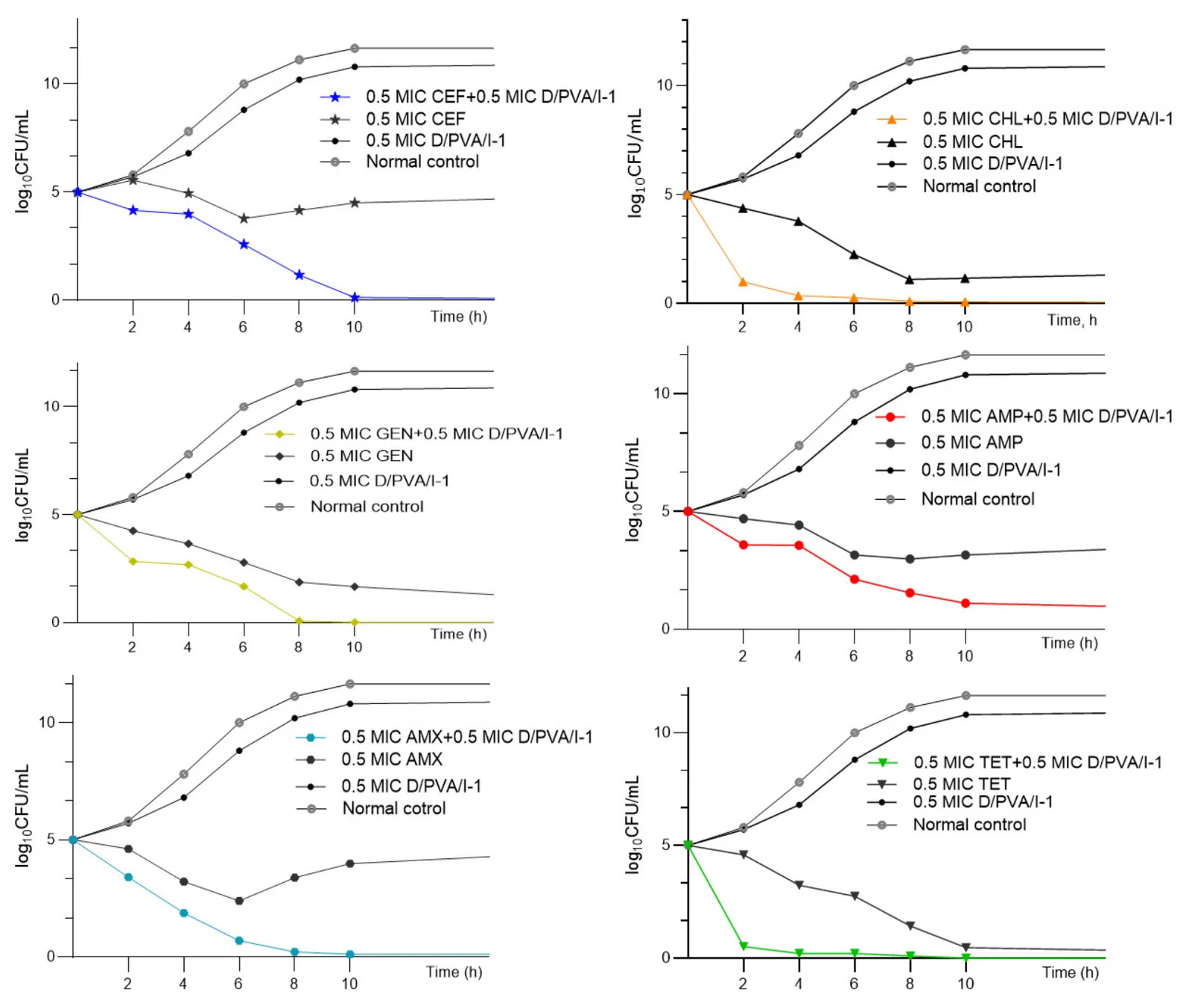
Time-kill curves for *E.coli* ATCC BAA-196 strain in presence of D/PVA/I-1 and AB (CEF, CHL, GEN, AMP, AMX, TET) alone or in combination, and a normal control (only medium without any drug) for 24 h. The figure is original artwork created by the authors using GraphPad Prism 5 (GraphPad Software, Inc.).

**Figure 5:**
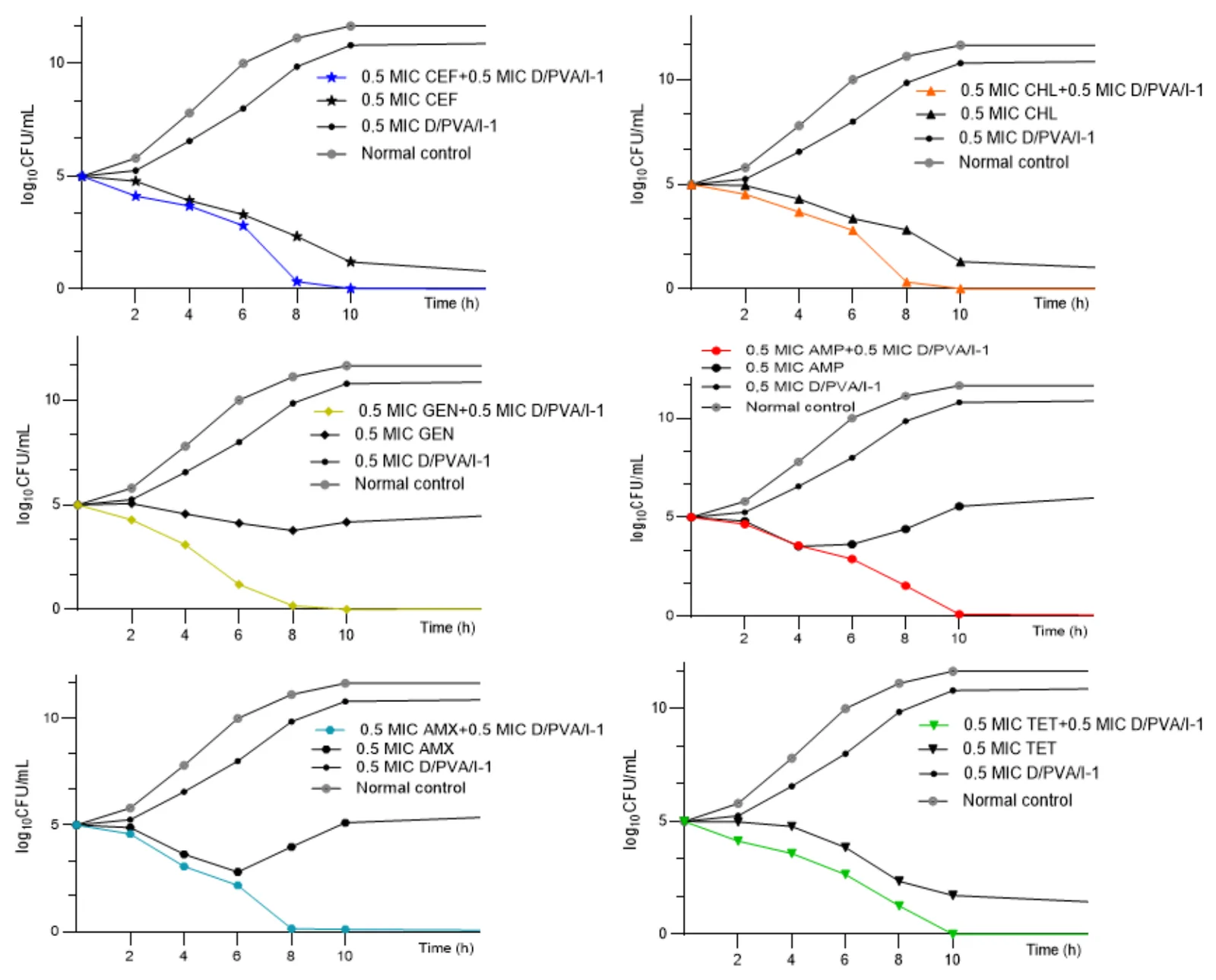
Time-kill curves for *E.coli* ATCC BAA-2523 strain in presence of D/PVA/I-1 and AB (CEF, CHL, GEN, AMP, AMX, TET) alone or in combination, and a normal control (only medium without any drug) for 24 h. The figure is original artwork created by the authors using GraphPad Prism 5 (GraphPad Software, Inc.).

**Figure 6:**
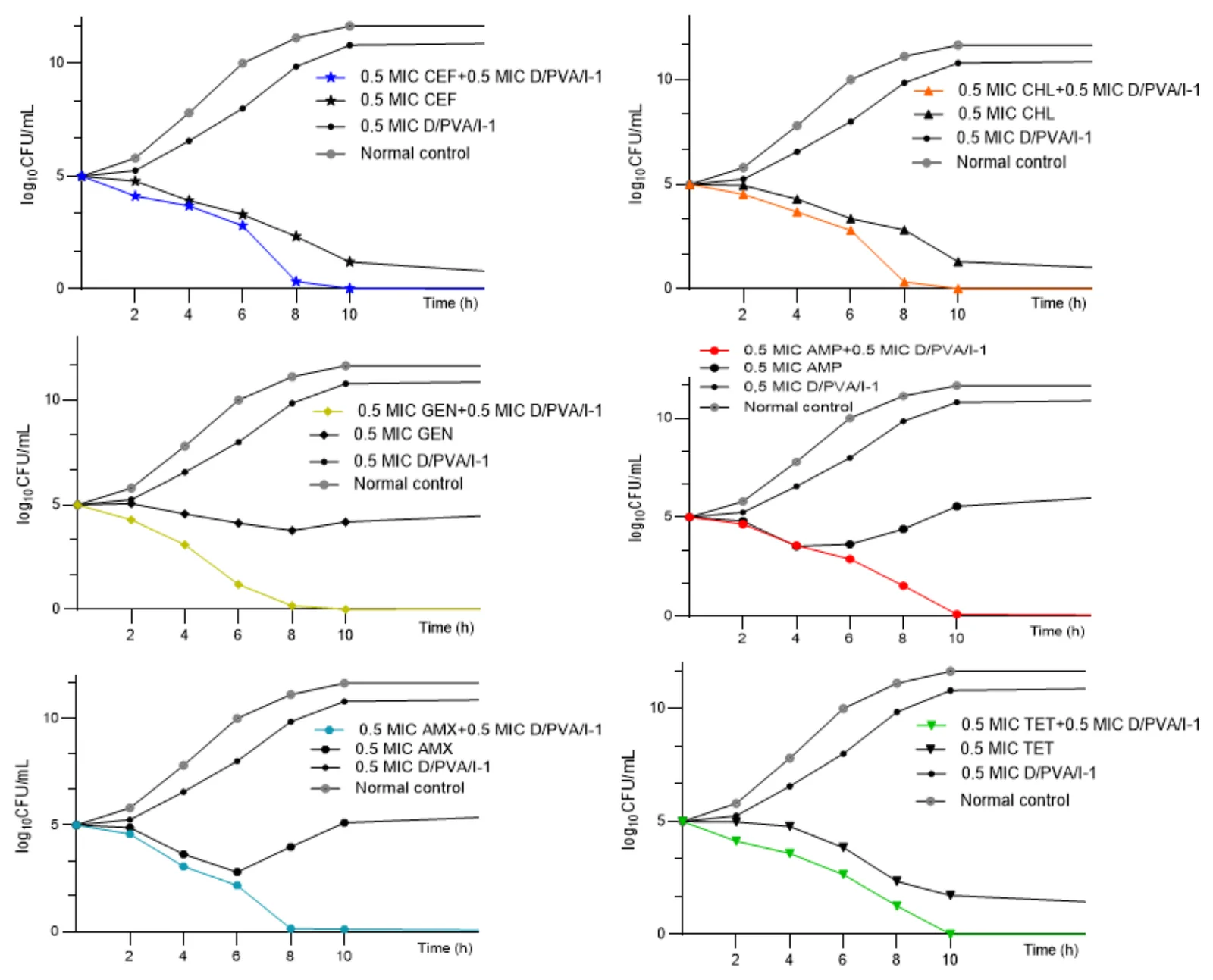
Time-kill curves for *K. pneumoniae* ATCC BAA-2524 strain in presence of D/PVA/I-1 and AB (CEF, CHL, GEN, AMP, AMX, TET) alone or in combination, and a normal control (only medium without any drug) for 24 h. The figure is original artwork created by the authors using GraphPad Prism 5 (GraphPad Software, Inc.).

**Figure 7:**
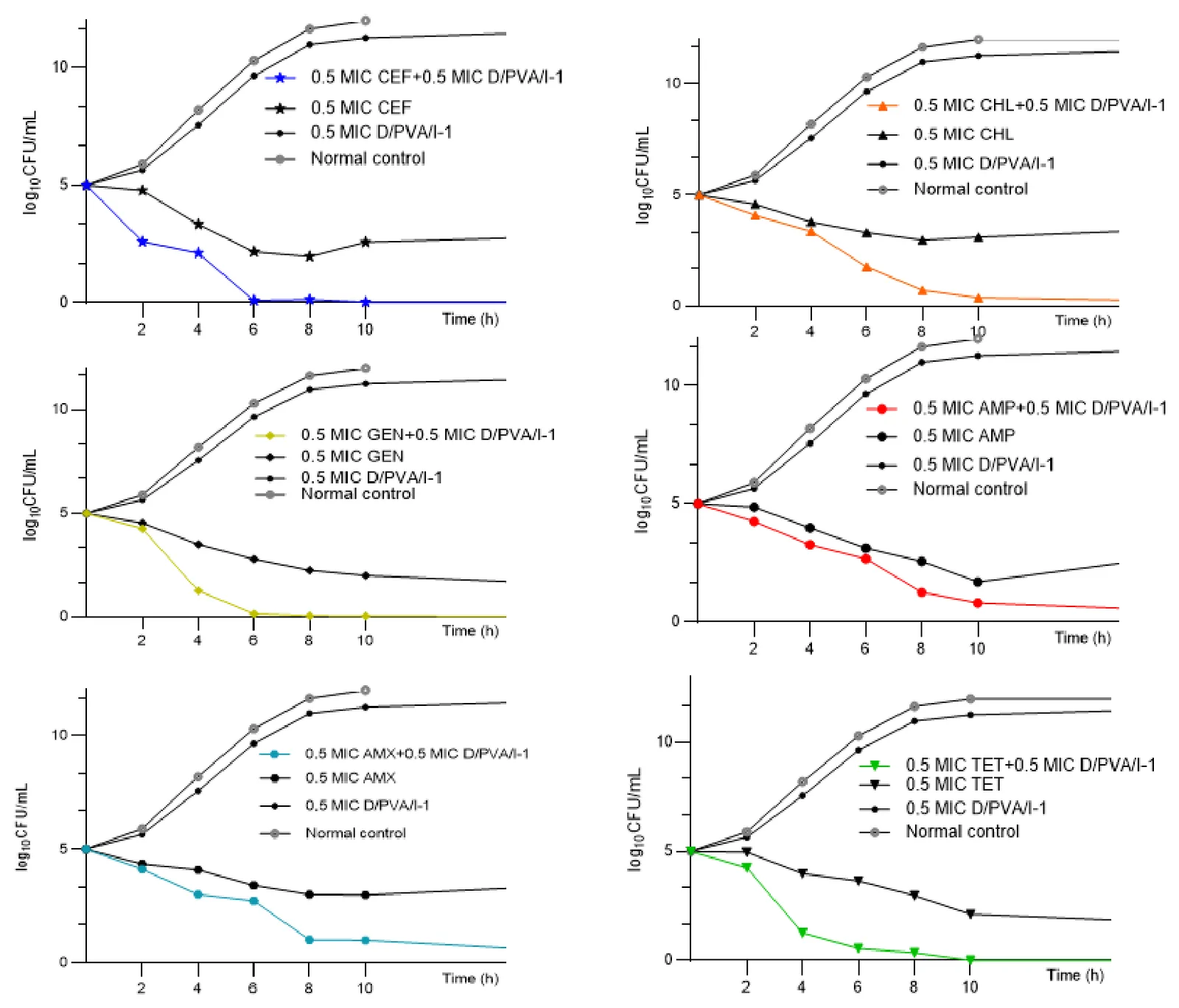
Time-kill curves for *K. pneumania* ATCC 700603 strain in presence of D/PVA/I-1 and AB (CEF, CHL, GEN, AMP, AMX, TET) alone or in combination, and a normal control (only medium without any drug) for 24 h. The figure is original artwork created by the authors using GraphPad Prism 5 (GraphPad Software, Inc.).

**Figure 8:**
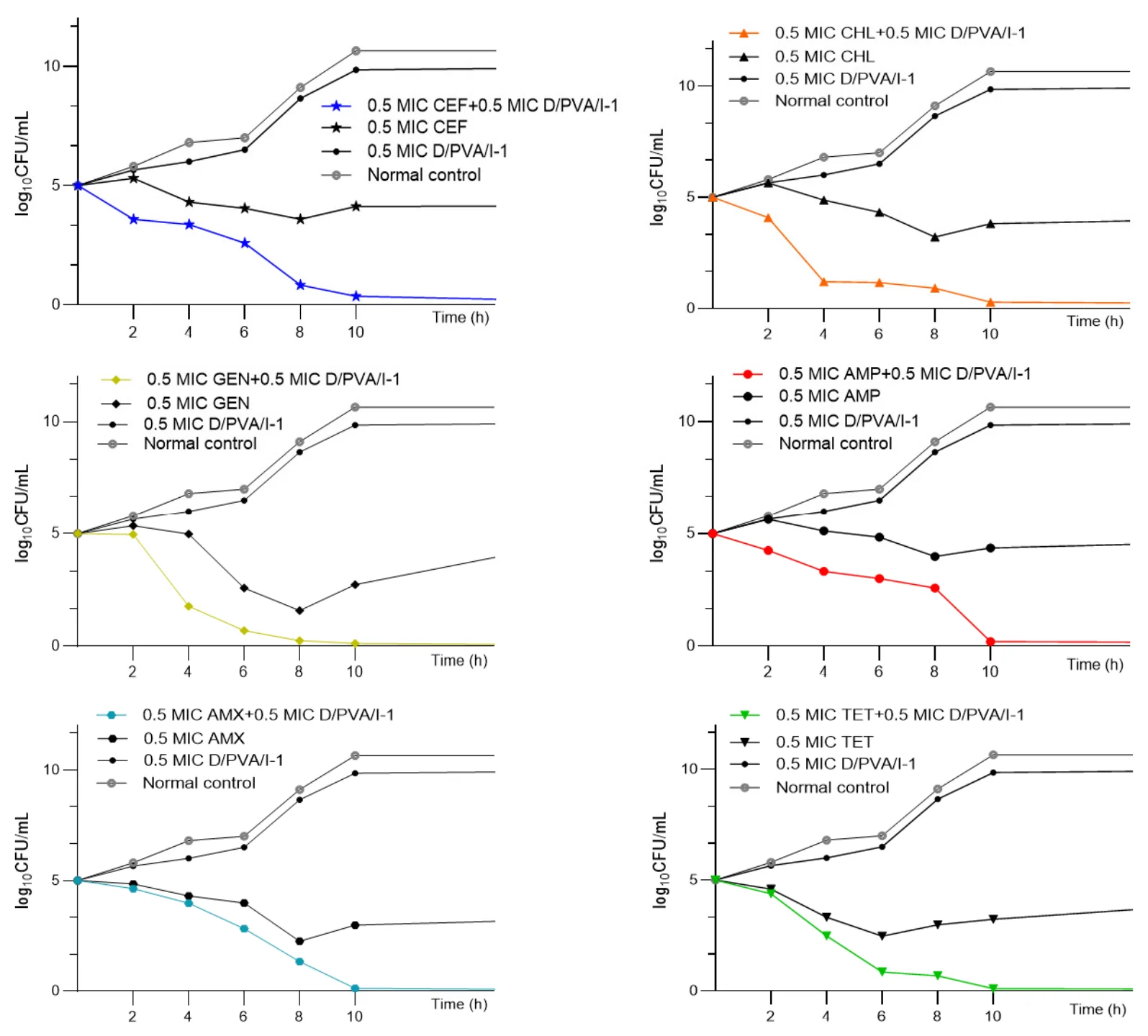
Time-kill curves for *A. baumannii* ATCC 1790 strain in presence of D/PVA/I-1 and AB (CEF, CHL, GEN, AMP, AMX, TET) alone or in combination, and a normal control (only medium without any drug) for 24 h. The figure is original artwork created by the authors using GraphPad Prism 5 (GraphPad Software, Inc.).

**Figure 9:**
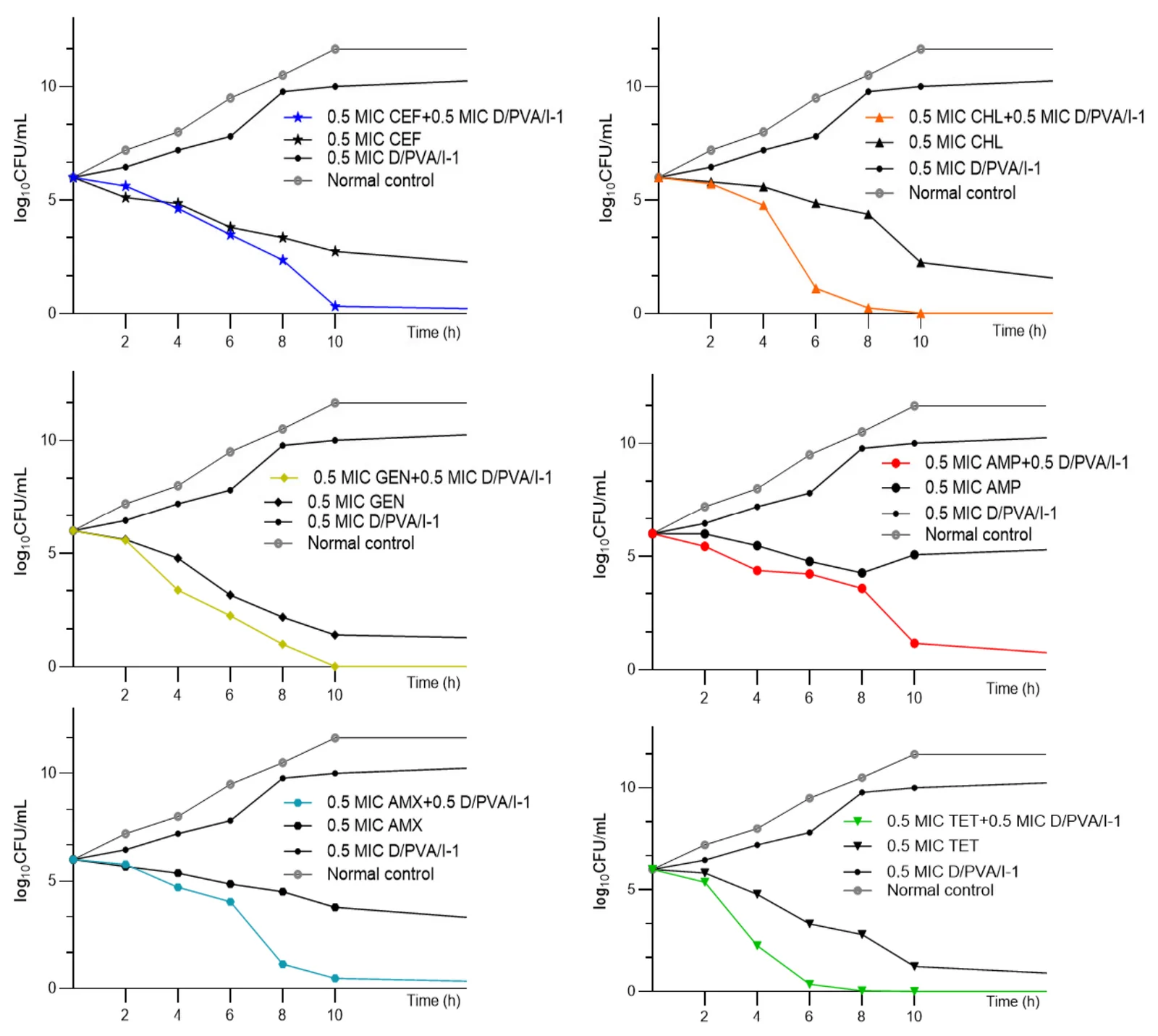
Time-kill curves for *S. pneumaniae* ATCC BAA-660 strain in presence of D/PVA/I-1 and AB (CEF, CHL, GEN, AMP, AMX, TET) alone or in combination, and a normal control (only medium without any drug) for 24 h. The figure is original artwork created by the authors using GraphPad Prism 5 (GraphPad Software, Inc.).

**Figure 10:**
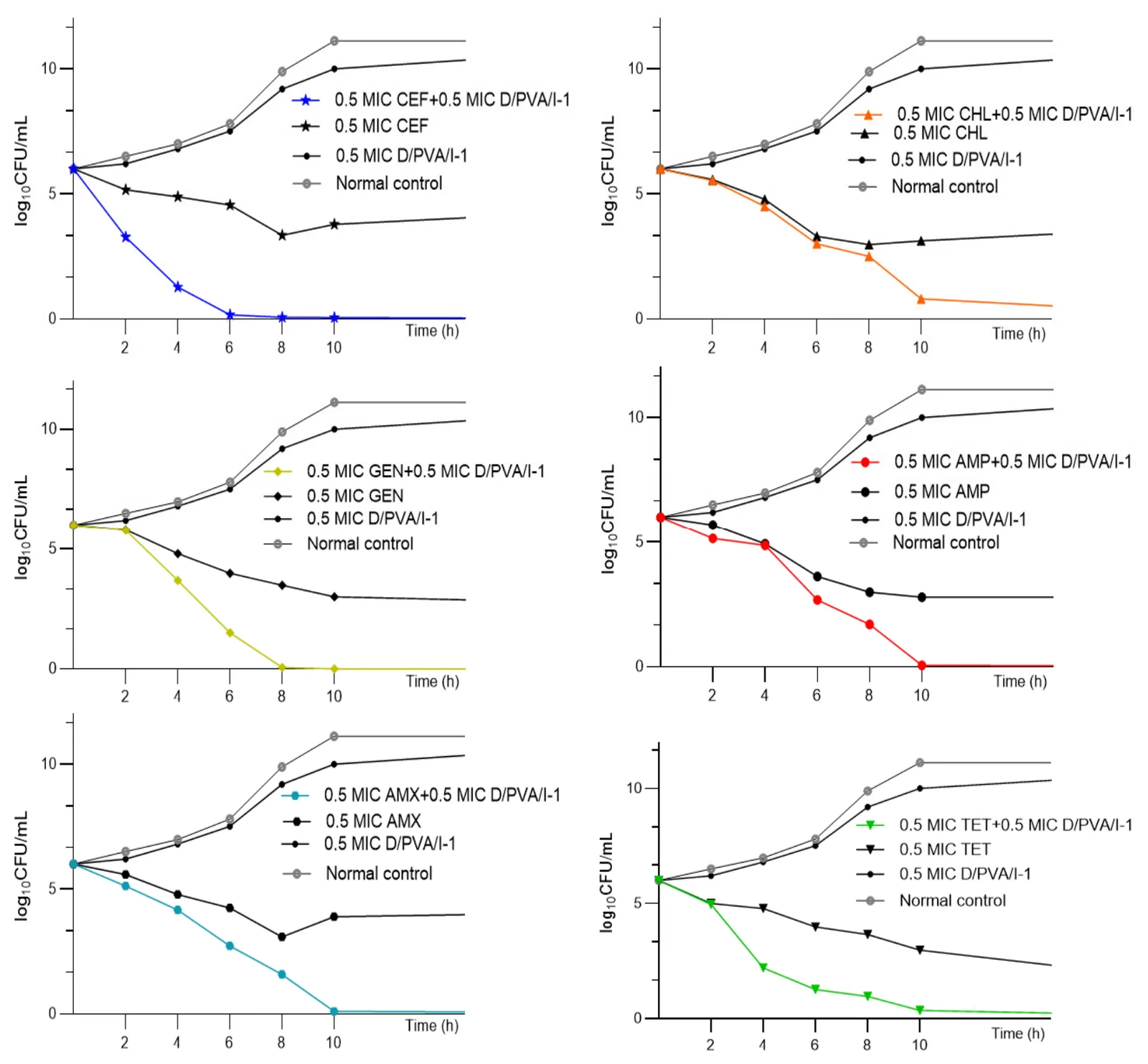
Time-kill curves for *Hemophilus influenzae* ATCC 33930 strain in presence of D/PVA/I-1 and AB (CEF, CHL, GEN, AMP, AMX, TET) alone or in combination, and a normal control (only medium without any drug) for 24 h. The figure is original artwork created by the authors using GraphPad Prism 5 (GraphPad Software, Inc.).

To evaluate the bactericidal kinetics of D/PVA/I-1 in combination with conventional antibiotics, time-kill analysis was performed against nine multidrug-resistant reference strains, including Gram-positive (*S. aureus* ATCC BAA-39, *S. aureus* ATCC 33591, *S. pneumoniae* ATCC BAA-660) and Gram-negative (*E. coli* ATCC BAA-196, *E. coli* ATCC BAA-2523, *K. pneumoniae* ATCC BAA-2524, *K. pneumoniae* ATCC 700603, *A. baumannii* ATCC BAA-1790, *H. influenzae* ATCC 33930) pathogens. Each strain was exposed to six antibiotics—ampicillin, amoxicillin, cefazolin, chloramphenicol, gentamicin, and tetracycline—administered both as monotherapy and in combination with D/PVA/I-1 at sub-MBC concentrations. CFU/mL was monitored at 0, 2, 4, 6, 8, and 10 h. Synergism was defined as a ≥2 log10 reduction in CFU/mL at 24 h compared to the most active single agent. The corresponding time-kill kinetics are shown in Figure 2, Figure 3, Figure 4, Figure 5, Figure 6, Figure 7, Figure 8, Figure 9 and Figure 10.

Monotherapy with ampicillin and amoxicillin failed to reduce bacterial counts meaningfully against *S. aureus* ATCC BAA-39, while cefazolin, chloramphenicol, gentamicin, and tetracycline produced partial bacteriostatic effects (CFU reduction to 1.5–2.5 log_10_ CFU/mL) without complete inhibition. Co-administration with D/PVA/I-1 markedly accelerated killing across all combinations: gentamicin achieved complete inhibition by 6 h, and chloramphenicol and cefazolin by 8–10 h. Notably, the ampicillin combination, despite complete monotherapy failure, reached full sterilization by 8–10 h, demonstrating the capacity of D/PVA/I-1 to restore activity against an intrinsically resistant antibiotic.

Figure 3 shows the time-kill data for *Staphylococcus aureus* ATCC 33591 treated with D/PVA/I-1 in combination with antibiotics.

Antibiotic monotherapy produced partial bacteriostatic or bactericidal activity across all agents, with CFU reductions ranging from approx. 1.0 to 4.0 log10 by 10 h, none achieving complete inhibition. Combination with D/PVA/I-1 enhanced the bactericidal activity of all six antibiotics tested. The most pronounced and rapid synergistic effect was observed with gentamicin, where CFU declined to approx. 1.5 log10 by 2 h and complete inhibition was achieved by 6–8 h, with curve divergence apparent from hour 1, indicative of marked time-dependent synergism. Chloramphenicol combination showed substantial synergy, with progressive CFU reduction from hour 1 and complete inhibition by 10 h. Cefazolin and amoxicillin combinations demonstrated partial synergism, with complete inhibition reached by 10 h and curve divergence from hours 4 and 2, respectively. The ampicillin combination also achieved complete inhibition by 10 h, with divergence from hour 6. Notably, the tetracycline combination exhibited synergistic interaction—complete inhibition by 8 h—in contrast to the indifferent response observed for *S. aureus* ATCC BAA-39, suggesting strain-specific modulation of antibiotic activity by D/PVA/I-1. The untreated control showed uninhibited growth throughout the observation period.

The time-kill results for *E. coli* ATCC BAA-196 are presented in Figure 4.

The dominant feature of monotherapy failure in this strain was not insufficient killing but instability of effect: ampicillin and cefazolin exhibited pronounced regrowth by 6–10 h, and amoxicillin was inactive throughout. D/PVA/I-1 addressed both dimensions simultaneously, accelerating killing for gentamicin and tetracycline (complete inhibition by 4–6 h) and abolishing regrowth entirely for the β-lactam agents, converting a rebound pattern into sustained bactericidal activity.

Figure 5 presents the time-kill data for the multidrug-resistant strain *Escherichia coli* ATCC BAA-2523 treated with D/PVA/I-1 in combination with ampicillin, amoxicillin, tetracycline, chloramphenicol, gentamicin, and cefazolin.

Antibiotic monotherapy produced variable bactericidal activity, with evidence of resistance-associated regrowth for several agents. Ampicillin monotherapy was largely ineffective, with CFU remaining stable or increasing slightly throughout the experiment. Amoxicillin and gentamicin monotherapy resulted in transient CFU reductions followed by marked regrowth by 8–10 h, reflecting multidrug resistance of the strain. Cefazolin, chloramphenicol, and tetracycline monotherapy achieved partial CFU reductions (3.5–4.5 log_10_) without complete inhibition. Combination with D/PVA/I-1 potentiated bactericidal activity across all six agents. The earliest synergistic effect was observed with cefazolin, with curve divergence from 1 h and complete inhibition by 8 h, consistent with pronounced time-dependent synergism. The gentamicin combination achieved complete inhibition by 6–8 h, with significant curve divergence from hour 2 and full suppression of the regrowth phenotype. Ampicillin and amoxicillin combinations demonstrated marked partial synergism, with complete inhibition by 10 h and complete abrogation of resistance-associated regrowth. Chloramphenicol combination reached complete inhibition by 8–10 h with moderate synergism. Tetracycline exhibited marginal synergism, with curves remaining in close proximity throughout the experiment. These results demonstrate that D/PVA/I-1 effectively restores antibiotic susceptibility and suppresses regrowth in a multidrug-resistant *E. coli* strain.

The time-kill results for *Klebsiella pneumoniae* ATCC BAA-2524 are presented in Figure 6.

Ampicillin monotherapy resulted in net bacterial recovery to approx. 5.5 log_10_ CFU/mL by 8–10 h, and amoxicillin produced an initial CFU increase upon exposure. Remaining antibiotics achieved partial reductions without sterilization. In combination with D/PVA/I-1, gentamicin achieved near-complete inhibition by 4 h and sterilization by 6–8 h. Most clinically relevant was the ampicillin result: D/PVA/I-1 not only halted regrowth but drove the combination to complete inhibition by 10 h, demonstrating restoration of penicillin-class activity against this MDR strain.

Figure 7 presents the time-kill data for the multidrug-resistant strain *Klebsiella pneumoniae* ATCC 700603 treated with D/PVA/I-1 in combination with ampicillin, amoxicillin, tetracycline, chloramphenicol, gentamicin, and cefazolin.

Antibiotic monotherapy produced partial and variable bactericidal activity. Cefazolin monotherapy exhibited a biphasic response—initial CFU reduction to approx. 0.5 log_10_ by 6 h followed by pronounced regrowth to 2.5 log_10_ by 8–10 h, indicative of a resistance-associated rebound. Amoxicillin and chloramphenicol monotherapy achieved modest reductions of approx. 2.0 log_10_, while ampicillin, gentamicin, and tetracycline yielded reductions of approx. 3.0–3.5 log_10_, none reaching complete inhibition. Combination with D/PVA/I-1 potentiated bactericidal activity across all agents. The most rapid synergistic effect was observed with gentamicin, with curve divergence from hour 1, complete inhibition achieved by 6 h, and effect sustained to 10 h. Tetracycline combination demonstrated pronounced synergism, with curve divergence from 2 h and complete inhibition by 6–8 h, contrasting with the absence of complete inhibition under monotherapy. Cefazolin combination fully abrogated the regrowth phenotype, achieving complete and sustained inhibition by 6 h, consistent with marked time-dependent synergism. Chloramphenicol and amoxicillin combinations reached complete inhibition by 8–10 h, with substantial curve divergence throughout the experiment. Ampicillin exhibited moderate synergism, with gradual curve divergence from hour 2 and CFU reduction to approx. 1.0 log_10_ by 10 h without complete inhibition. Compared to *K. pneumoniae* ATCC 2524, this strain demonstrated a broader spectrum of synergistic interactions, particularly for tetracycline and chloramphenicol combinations.

Figure 8 presents the time-kill data for *Acinetobacter baumannii* ATCC BAA-1790 treated with D/PVA/I-1 in combination with ampicillin, amoxicillin, tetracycline, cefazolin, gentamicin, and chloramphenicol.

Monotherapy produced the most limited bactericidal activity across the entire dataset, with several agents—chloramphenicol and gentamicin—exhibiting paradoxical initial CFU increases at 2 h before partial reduction, and ampicillin remaining entirely inactive. Despite this challenging baseline, D/PVA/I-1 achieved synergistic or partially synergistic interactions with all six antibiotics. Chloramphenicol combination produced the steepest killing curve (complete inhibition by 8–10 h), and gentamicin fully reversed its biphasic monotherapy pattern, reaching sustained sterilization by 6 h. Given the severely limited therapeutic options for *A. baumannii* in clinical settings, these findings are of particular relevance.

Figure 9 presents the time-kill data for *Streptococcus pneumoniae* ATCC BAA-660 treated with D/PVA/I-1 in combination with antibiotics.

Tetracycline monotherapy produced the fastest initial killing in the dataset (approx. 5.0 log_10_ CFU/mL reduction by 2 h) but plateaued without achieving complete inhibition, consistent with a tolerant subpopulation. D/PVA/I-1 preserved the rapid early kinetics while closing the gap to full sterilization by 6 h, suggesting targeted activity against the tolerant fraction that escapes monotherapy. Chloramphenicol combination achieved complete inhibition 2–4 h earlier than any monotherapy agent, while ampicillin, near-inactive alone, reduced CFU to approx. 0.5 log_10_ CFU/mL by 10 h in combination.

Figure 10 presents the time-kill data for *Haemophilus influenzae* ATCC 33930 treated with D/PVA/I-1 in combination with antibiotics.

Monotherapy produced a narrow band of partial activity across all six agents (CFU reduction 2.5–3.5 log_10_ CFU/mL), with amoxicillin the sole agent exhibiting frank regrowth by 8–10 h. The D/PVA/I-1 combinations clearly differentiated outcomes that monotherapy could not: cefazolin achieved complete inhibition by 6 h (4 h ahead of any monotherapy agent), while ampicillin and amoxicillin fully overcame stabilization and regrowth, respectively. Chloramphenicol and tetracycline showed moderate synergism without full sterilization. Together with data from all nine strains, these results confirm consistent antibiotic-potentiating activity of D/PVA/I-1 across both Gram-positive and Gram-negative MDR pathogens.

**Figure 11:**
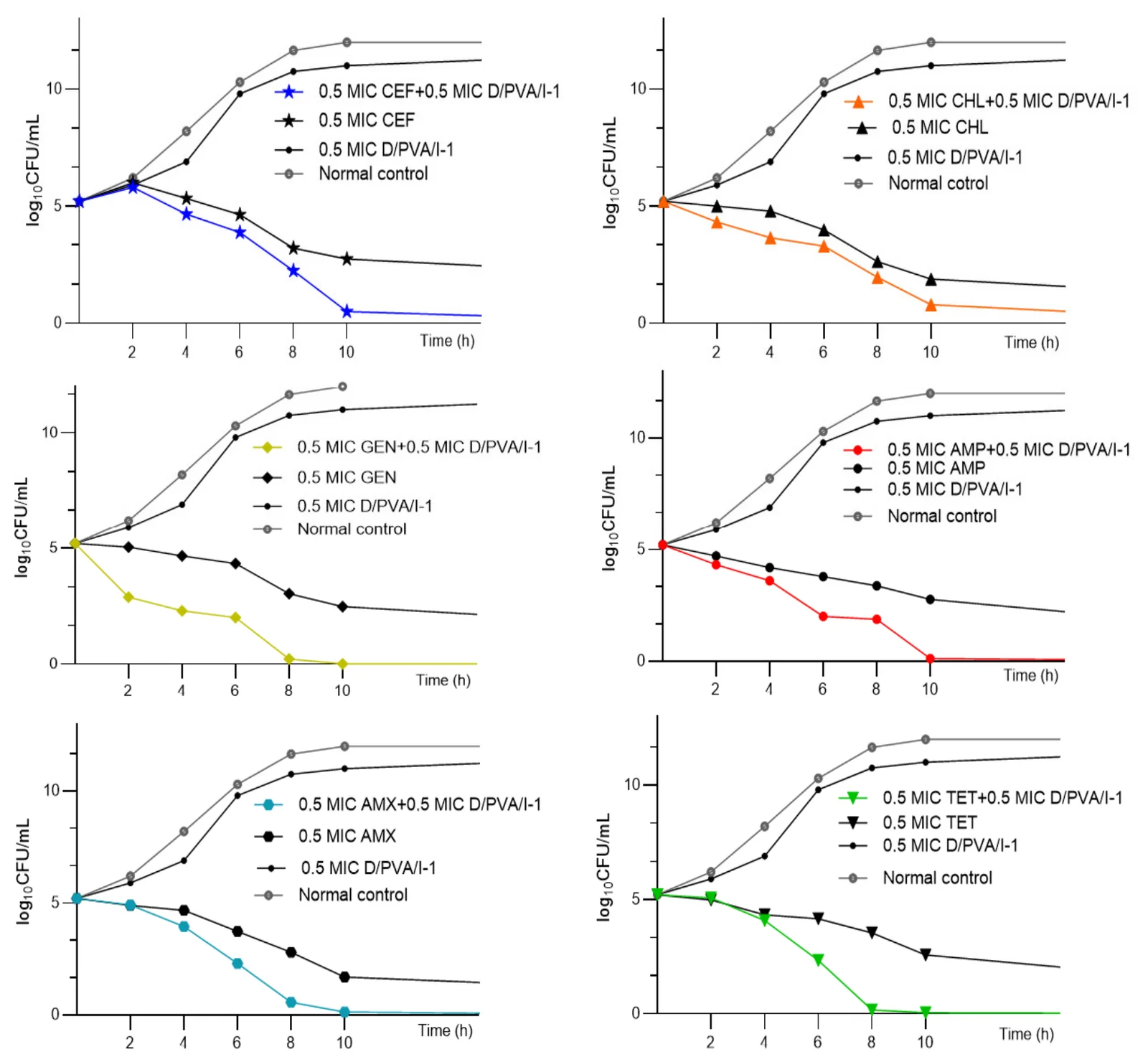
Time-kill curves for *P. aeruginosa* SCAID PHRX1-2019 clinical isolate in presence of D/PVA/I-1 and AB (CEF, CHL, GEN, AMP, AMX, TET) alone or in combination, and a normal control (only medium without any drug) for 24 h. The figure is original artwork created by the authors using GraphPad Prism 5 (GraphPad Software, Inc.).

Figure 11 presents the time-kill data for *P. aeruginosa* SCAID PHRX1-2019 treated with D/PVA/I-1 in combination with antibiotics.

Time-kill assays against *P. aeruginosa* SCAID PHRX1-2019 demonstrated that D/PVA/I-1 potentiated the bactericidal activity of all six antibiotics tested at 0.5× MIC (Figure 11). The untreated growth control proliferated continuously, reaching approximately 10^12^–10^13^ CFU/mL by 10 h, while each antibiotic alone at 0.5× MIC produced only a modest bacteriostatic effect, with viable counts remaining largely stable or declining slightly over the observation period. In combination with 0.5× MIC D/PVA/I-1, however, all six regimens produced a marked, progressive decline in viable counts. The GEN and TET combinations were the most rapidly bactericidal, reducing counts below the detection limit by 8–10 h. The AMX and CEF combinations similarly drove bacterial density to near-undetectable levels by 10 h, whereas the AMP and CHL combinations achieved substantial, though somewhat less complete, reductions over the same interval. In every case, the combination curves diverged from the corresponding monotherapy curves by more than 2 log_10_ CFU/mL, indicating a synergistic bactericidal interaction between D/PVA/I-1 and each antibiotic against this MDR *P. aeruginosa* strain.

**Figure 12:**
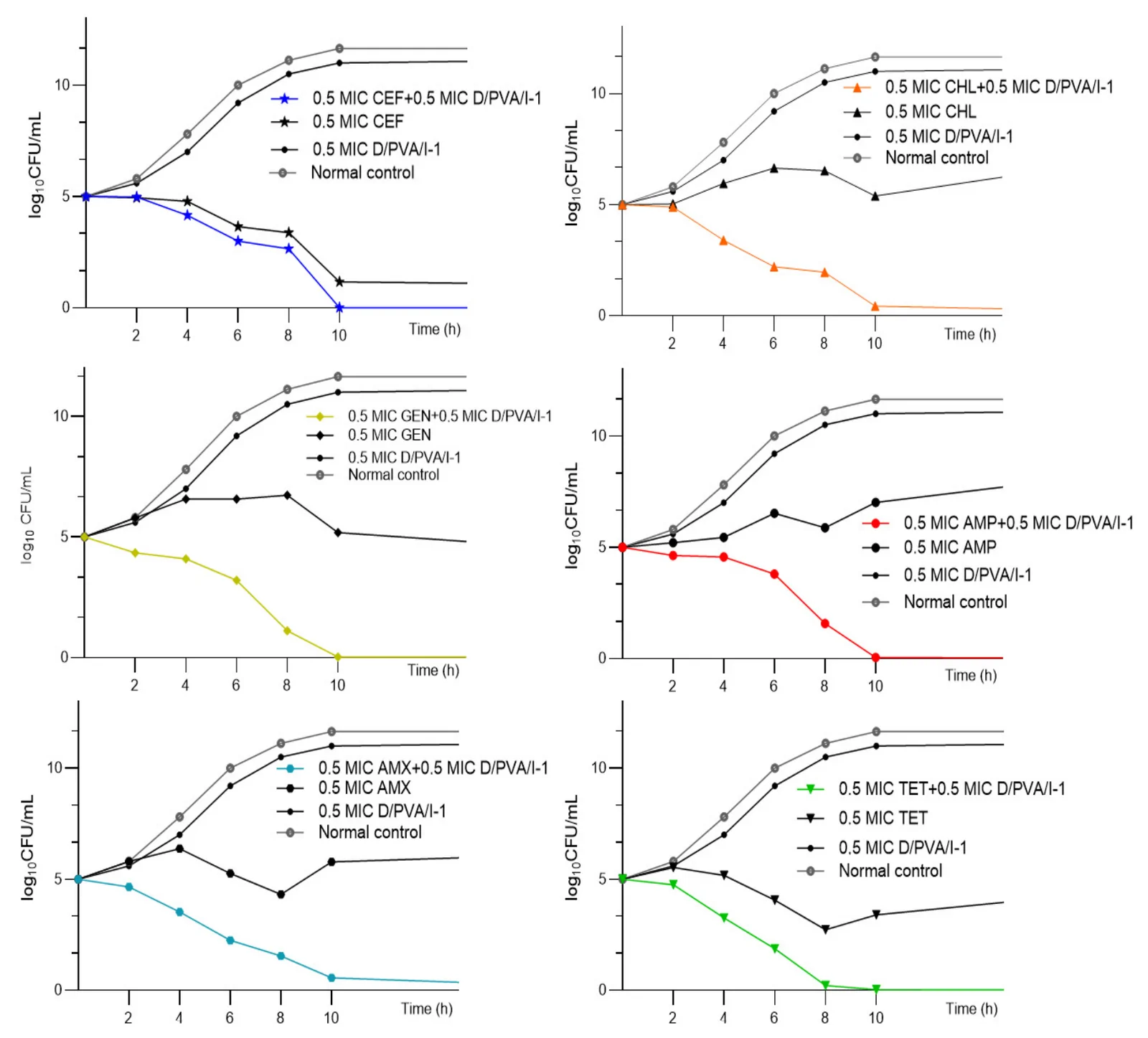
Time-kill curves for *E. coli* SCAID WND1-2021 clinical isolate in presence of D/PVA/I-1 and AB (CEF, CHL, GEN, AMP, AMX, TET) alone or in combination, and a normal control (only medium without any drug) for 24 h. The figure is original artwork created by the authors using GraphPad Prism 5 (GraphPad Software, Inc.).

Figure 12 presents the time-kill data for *E. coli* SCAID WND1-2021 treated with D/PVA/I-1 in combination with antibiotics.

Against *E. coli* SCAID WND1-2021, the untreated growth control proliferated steadily throughout the 10 h period, reaching approximately 10^11^–10^12^ CFU/mL. Antibiotic monotherapy at 0.5× MIC generally failed to suppress bacterial growth: CHL, GEN, AMP, AMX, and TET alone all permitted an initial rise in viable counts during the first 2–4 h before counts plateaued or declined only modestly by 10 h, indicating limited bacteriostatic activity of these agents at sub-inhibitory concentration against this strain. CEF monotherapy was somewhat more effective, producing a gradual decline over the full time course. Combination with 0.5× MIC D/PVA/I-1 markedly enhanced antibacterial activity for all six antibiotics. The GEN and TET combinations showed a delayed but sharp bactericidal effect, with counts dropping precipitously after 6 h to reach the detection limit by 8–10 h. The AMP combination tracked closely with its monotherapy counterpart through 6 h before undergoing a rapid decline to below the detection limit by 10 h. The CEF, CHL, and AMX combinations exhibited a more gradual, progressive decline from the outset, reaching near-undetectable or markedly reduced counts (≥3–4 log_10_ CFU/mL reduction) by 10 h. In each case, the combination curve diverged substantially from the corresponding monotherapy curve, consistent with a synergistic enhancement of bactericidal activity when D/PVA/I-1 was combined with each antibiotic against this MDR *E. coli* strain. Collectively, these findings, together with data across all nine strains tested, confirm the broad synergistic potential of D/PVA/I-1 against both Gram-positive (*S. aureus*, *S. pneumoniae*) and Gram-negative (*E. coli*, *K. pneumoniae*, *H. influenzae*, *A. baumannii*) multidrug-resistant pathogens.

### 2.2. Checkerboard Assay Results

To quantitatively assess the interaction between D/PVA/I-1 and conventional antibiotics, checkerboard assays were performed against five multidrug-resistant reference strains: *S. aureus* ATCC 33591, *S. aureus* ATCC BAA-39, *E. coli* ATCC BAA-196, *K. pneumoniae* ATCC BAA-2524, and *A. baumannii* ATCC BAA-1790. Each strain was tested against nine antibiotics—ceftriaxone, levofloxacin, gentamicin, tetracycline, azithromycin, amoxicillin, ampicillin, chloramphenicol, and doxycycline—in pairwise combination with D/PVA/I-1. The results are summarized in Table 1.

The ability of D/PVA/I-1 to potentiate the activity of conventional antibiotics was evaluated against five multidrug-resistant reference strains using the fractional inhibitory concentration index (FICI). Across the 45 antibiotic–D/PVA/I-1 combinations tested, synergistic or partially synergistic interactions predominated, with no antagonistic effects observed.

For the Gram-positive strains, *Staphylococcus aureus* ATCC 33591 exhibited synergistic interactions with seven of nine antibiotics tested (77.8%), with FICI values ranging from 0.313 to 0.500. The strongest effect was observed for chloramphenicol (FICI = 0.313), corresponding to a 16-fold reduction in MIC. *S. aureus* ATCC BAA-39 demonstrated synergy with five of nine antibiotics (55.6%), while the remaining combinations showed partial synergy (FICI = 0.563–0.750).

Among Gram-negative strains, *Escherichia coli* ATCC BAA-196 displayed synergistic interactions with levofloxacin and tetracycline (FICI = 0.390), whereas the remaining antibiotics exhibited partial synergy (FICI = 0.510–0.760). Similarly, *Klebsiella pneumoniae* ATCC BAA-2524 showed synergy only with gentamicin and chloramphenicol (FICI = 0.390 and 0.397, respectively), while all other combinations resulted in partial synergy. In *Acinetobacter baumannii* ATCC 1790, synergy was observed with levofloxacin, gentamicin, amoxicillin, and ampicillin (FICI = 0.375–0.500), whereas ceftriaxone, tetracycline, and doxycycline displayed partial synergy, and azithromycin and chloramphenicol showed additive effects (FICI = 1.0).

Overall, 20 of 45 combinations (44.4%) demonstrated synergy (FICI ≤ 0.5), 22 combinations (48.9%) showed partial synergy (0.5 < FICI ≤ 0.75), and three combinations (6.7%) exhibited additive effects (FICI = 1.0). No antagonistic interactions were detected. Synergistic effects were more frequent in Staphylococcus spp. (66.7% of combinations) than in Gram-negative pathogens (33.3%), indicating that D/PVA/I-1 enhances antibiotic efficacy across diverse resistance phenotypes, with particularly strong potentiation against methicillin-resistant *S. aureus* strains.

### 2.3. Membrane Permeability Assay Results

To investigate the mechanism underlying the antibiotic-potentiating activity of D/PVA/I-1, its effect on bacterial membrane permeability was evaluated using a crystal violet uptake assay. CTAB and polymyxin B were included as positive controls, and experiments were conducted against both susceptible reference strains (*S. aureus* ATCC 6538-p and *K. pneumoniae* ATCC 10031) and multidrug-resistant strains (*S. aureus* ATCC BAA-33591 and *K. pneumoniae* ATCC 2524).

**Figure 13:**
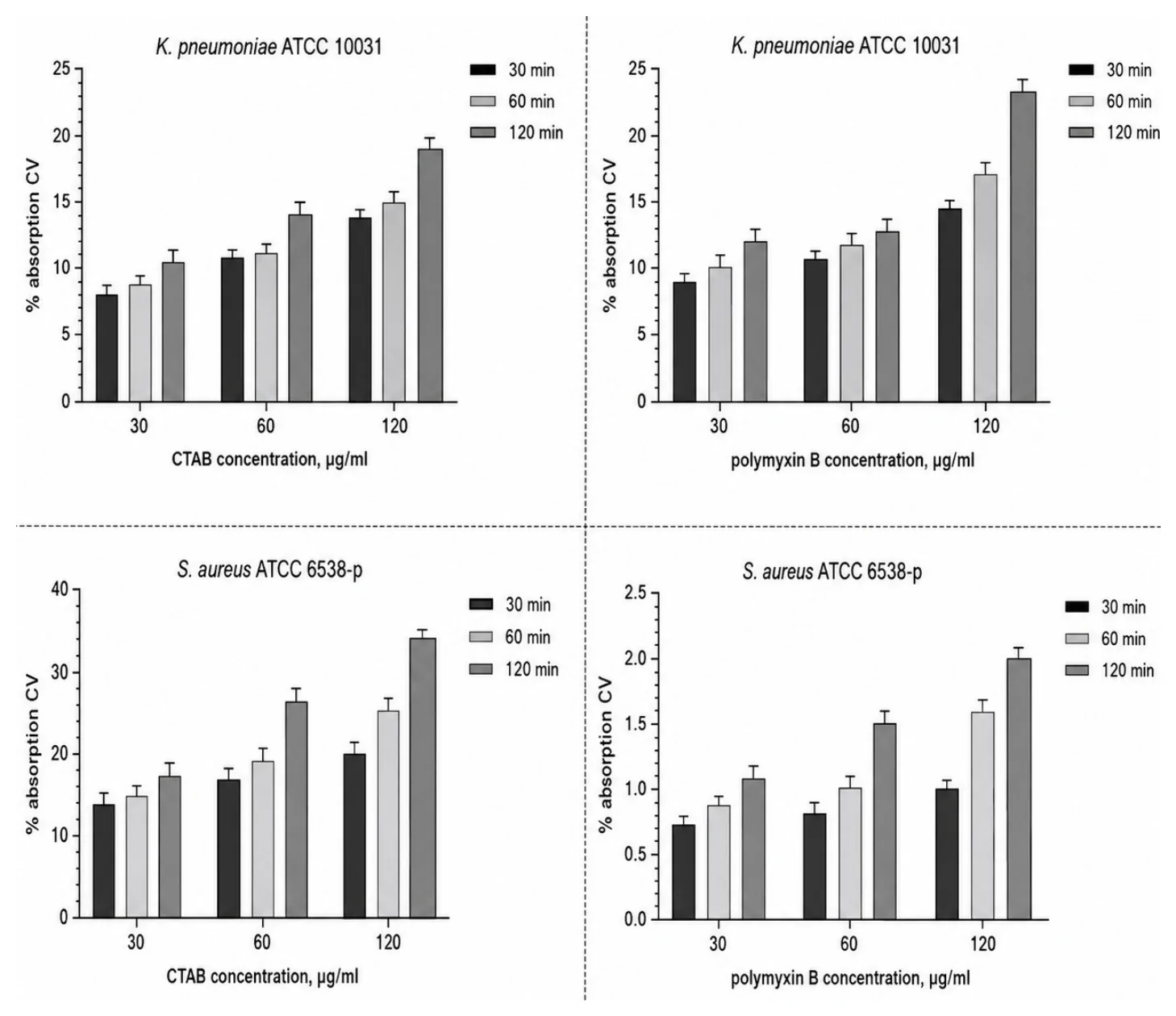
Change in bacterial membrane permeability in the presence of CTAB and Polymyxin B, expressed as crystal violet (CV) uptake (%) = (OD of sample/OD of CV solution) × 100, where OD is the optical density measured at 590 nm.

The presented data reflect the dynamics of crystal violet (CV) absorption under the influence of CTAB and Polymyxin B on *S. aureus* ATSS 6538-p and *K. pneumoniae* ATCC 10031 (Table 2, Figure 13).

CTAB induced a pronounced, dose- and time-dependent increase in crystal violet absorption in both species, with the effect being consistently stronger against *S. aureus* than *K. pneumoniae* across all concentrations and time points, reaching 33.01% and 19.54% absorption, respectively, at 120 μg/mL after 120 min. This difference is consistent with the structural advantage conferred by the outer membrane lipopolysaccharide layer in Gram-negative bacteria, which limits the access of cationic membrane-active agents to the cytoplasmic membrane. Polymyxin B displayed the expected species selectivity: negligible activity against *S. aureus* ATCC 6538-p (maximum 2.98% at 120 μg/mL, 120 min) and substantial membrane disruption in *K. pneumoniae* ATCC 10031 (22.98% under the same conditions), confirming the validity of the assay system.

To evaluate the membrane permeabilization effect of D/PVA/I-1, its minimum bactericidal concentration (MBC) was first determined against each test strain, and the corresponding sub-inhibitory concentrations (1/2 MBC and 1/4 MBC) were subsequently used in the membrane permeability assays (Table 3).

The MBC of D/PVA/I-1 was first determined for each test strain (Table 3). Against *Staphylococcus aureus* ATCC 6538-P, the MBC was 0.16 μg/mL, whereas the methicillin-resistant strain *S. aureus* ATCC BAA-33591 required a two-fold higher concentration (0.33 μg/mL) for a bactericidal effect. A similar pattern was observed for *Klebsiella pneumoniae*: the reference strain ATCC 10031 showed an MBC of 0.34 μg/mL, while the multidrug-resistant strain ATCC BAA-2524 required 0.68 μg/mL. In all cases, MDR strains required approximately twice the MBC observed for their corresponding reference strains. The determined MBC values were used to calculate the sub-inhibitory concentrations (1/2 MBC and 1/4 MBC) applied in the subsequent membrane permeabilization assays.

**Figure 14:**
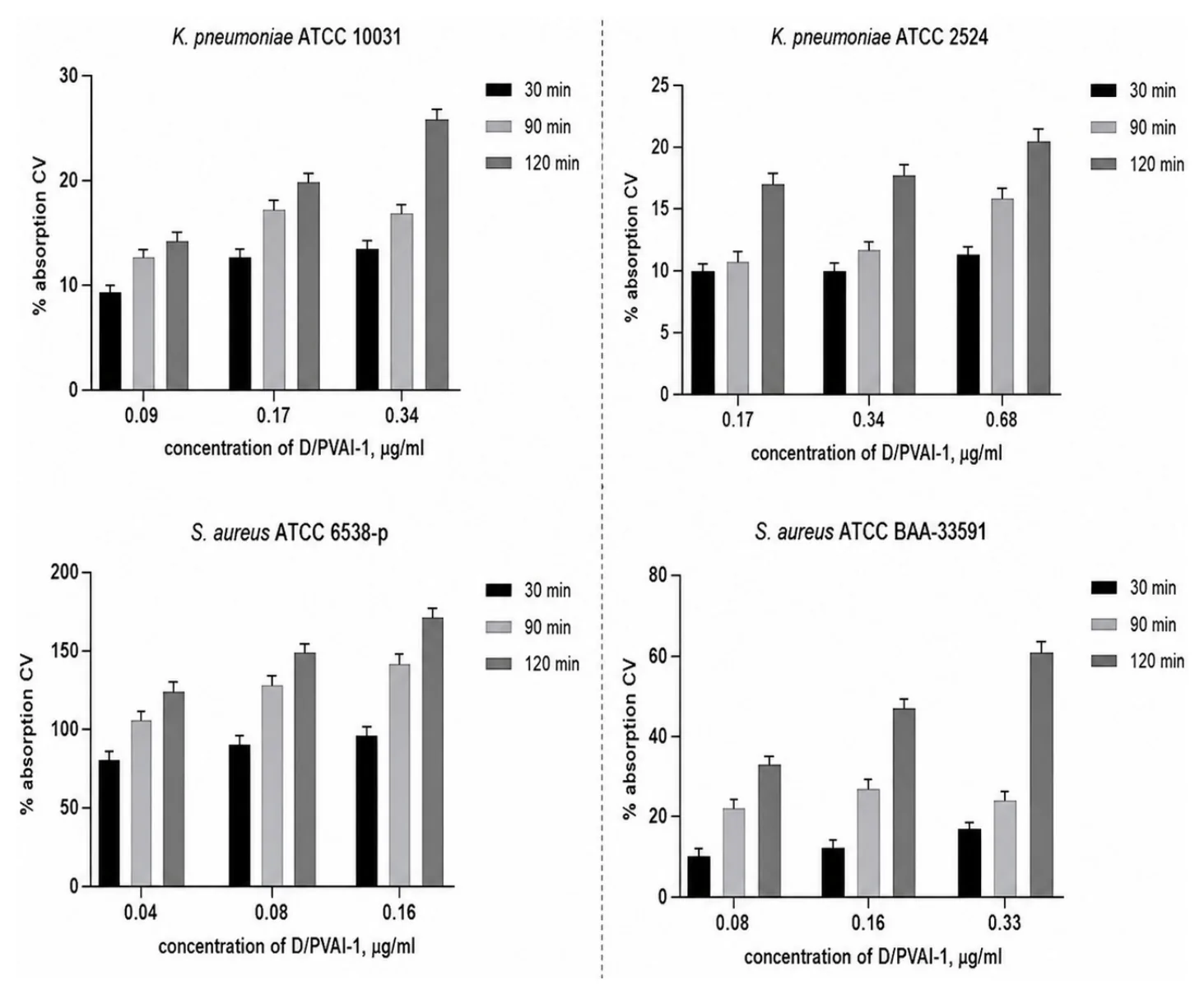
Change in permeability of bacterial membranes in the presence of D/PVA/I-1, expressed as crystal violet (CV) uptake (%) = (OD of sample/OD of CV solution) × 100, where OD is the optical density measured at 590 nm.

D/PVA/I-1 increased membrane permeability in all four strains tested in a dose- and time-dependent manner (Table 4, Figure 14). The effect was most pronounced against the susceptible *S. aureus* ATCC 6538-p, where crystal violet absorption reached 170.67% at the MBC concentration (1.95 μg/mL) after 120 min, values substantially exceeding those produced by CTAB at far higher concentrations, indicating potent membranotropic activity. Against the multidrug-resistant *S. aureus* ATCC BAA-33591, the effect was markedly attenuated: absorption reached 59.67% at the MBC (3.95 μg/mL) after 120 min, approximately 2.9-fold lower than in the susceptible strain under equivalent conditions. A similar pattern was observed for *K. pneumoniae*: the susceptible strain ATCC 10031 showed 25.33% absorption at MBC (3.91 μg/mL) after 120 min, while the resistant strain ATCC 2524 reached only 20.0% at its higher MBC (7.82 μg/mL). Across all strains, the sharpest increase in permeability occurred between 30 and 90 min of exposure, after which the rate of increase slowed, suggesting a rapid initial phase of membrane disruption followed by a plateau.

Taken together, these data indicate that membrane disruption is a component of the antibacterial mechanism of D/PVA/I-1, and that reduced membrane permeability in resistant strains, likely attributable to modified lipopolysaccharide composition, altered porin expression, or enhanced efflux, partially accounts for their higher MBC values. The capacity of D/PVA/I-1 to increase membrane permeability even in resistant strains provides a mechanistic basis for its observed ability to potentiate antibiotic activity, as enhanced membrane disruption may facilitate intracellular antibiotic accumulation and thereby overcome resistance mechanisms dependent on reduced drug uptake.

## 3. Discussion

The present study evaluated the antibiotic-potentiating activity of the dextrin/polyvinyl alcohol/iodine complex (D/PVA/I-1) against a panel of clinically relevant multidrug-resistant reference strains using a suite of complementary in vitro methods: the checkerboard assay with FICI calculation, bactericidal time-kill kinetics analysis, and the crystal violet membrane permeability assay. The results demonstrate that D/PVA/I-1 functions as a broad-spectrum antibiotic potentiator, exhibiting synergistic or partially synergistic interactions across a wide range of antibiotic classes and pathogen types.

The overall distribution of FICI values: 44.4% synergistic combinations (FICI ≤ 0.5), 48.9% partially synergistic (0.5 < FICI ≤ 0.75), and 6.7% additive (FICI = 1.0), is consistent with the profile reported for established membrane-active potentiators in the literature. Potentiators (antibiotic adjuvants) that disrupt membrane permeability at sub-MIC concentrations are known to sensitize resistant strains to structurally diverse antibiotic classes by overcoming reduced drug uptake and active efflux—two primary determinants of intrinsic resistance in Gram-negative bacteria [9,10]. The complete absence of antagonism across all combinations is a particularly favorable outcome, as antagonistic interactions represent a recognized clinical risk when combining bacteriostatic and bactericidal agents and can diminish the overall therapeutic effect of combination regimens.

The most pronounced potentiating effect was observed against Gram-positive pathogens, particularly the two MRSA strains of *Staphylococcus aureus*: synergy was recorded in 77.8% of combinations for *S. aureus* ATCC 33591 and in 55.6% for *S. aureus* ATCC BAA-39. The FICI value of 0.313 obtained for the D/PVA/I-1-chloramphenicol combination against *S. aureus* ATCC 33591, corresponding to a 16-fold reduction in MIC, is consistent with the magnitude of potentiation described for other membrane-targeting adjuvants against MRSA. For instance, alpha-mangostin derivatives and emodin have been shown to restore beta-lactam activity against MRSA through a combination of membrane disruption and efflux system inhibition [4,7]. The data obtained for D/PVA/I-1 suggest an analogous mechanism: the 8–16-fold MIC reductions observed across multiple antibiotic classes are difficult to explain through a single-target interaction and are more consistent with a non-specific membrane permeability event that facilitates intracellular drug accumulation. The selective potentiation of beta-lactam activity is of particular translational interest, given that this antibiotic class has been rendered largely ineffective against MRSA through the acquisition of PBP2a, and that such potentiation is conceptually aligned with resensitization strategies exemplified by beta-lactamase inhibitor combinations such as ceftazidime–avibactam [6].

Among Gram-negative pathogens, the potentiating activity of D/PVA/I-1 was comparatively attenuated, yet retained clinical relevance. Against *Escherichia coli* ATCC BAA-196 and *Klebsiella pneumoniae* ATCC BAA-2524, synergistic combinations were less frequent (22.2% and 22.2%, respectively), with the majority of interactions falling within the partial synergy range. This pattern is consistent with the structural barrier imposed by the lipopolysaccharide (LPS) layer of the Gram-negative outer membrane, which restricts the access of membrane-active agents to the cytoplasmic membrane and constitutes the principal determinant of intrinsic resistance to cationic and amphiphilic compounds [11,12]. The fact that synergistic FICI values were nonetheless achieved for gentamicin and chloramphenicol against *K. pneumoniae* ATCC BAA-2524 indicates that D/PVA/I-1 can partially overcome this barrier at the tested concentrations, likely through partial outer membrane destabilization sufficient to enhance intracellular accumulation of structurally diverse antibiotics. An analogous mechanism has been proposed for amphiphilic alpha-hydrazide acid derivatives, which increase membrane fluidity and permeability without pronounced structural damage and display synergy with tetracycline against *E. coli* and with ciprofloxacin against resistant strains [13]. Notably, this capacity for partial membrane destabilization appears to be a shared feature of iodine-containing coordination complexes more broadly: the study of amino acid iodine complexes KC-270 and KC-271 against *S. aureus* BAA-39 and *E. coli* BAA-196 demonstrated that KC-270 most markedly enhanced the activity of a range of antibiotics when introduced during the lag phase of bacterial growth, in some cases fully restoring susceptibility, whereas KC-271 exhibited a weaker and more limited effect [14]. The results obtained in the present study for D/PVA/I-1 are consistent with this trend and, taken together with the partial synergy data for Gram-negative strains, confirm the promise of iodine-containing complexes as a class of broad-spectrum adjuvant agents capable of modulating membrane permeability across structurally distinct bacterial envelopes.

Time-kill kinetics analysis provided dynamic evidence of D/PVA/I-1–antibiotic interactions beyond the static endpoint assessment of the checkerboard assay. Two distinct pharmacodynamic phenotypes were consistently observed across all tested strains. First, D/PVA/I-1 accelerated bactericidal kinetics: in the majority of combinations, complete growth suppression (sterilization endpoint) was achieved 2–6 h earlier than with the most active agent in monotherapy, indicating that the potentiating effect operates throughout the concentration-time profile rather than being confined to a single concentration threshold. Second, and potentially of greater clinical relevance, D/PVA/I-1 suppressed the regrowth phenomenon—the partial recovery of bacterial populations observed during monotherapy, which was particularly pronounced for beta-lactam agents against *E. coli* ATCC BAA-196 and *E. coli* ATCC BAA-2523. This regrowth pattern, reflecting the selection of tolerant or persistently resistant subpopulations under single-agent pressure, is a recognized predictor of therapeutic failure in MDR infections [15]. The ability of D/PVA/I-1 to completely abolish regrowth in combination with beta-lactams suggests that its mechanism of action targets a cellular component distinct from those exploited by the antibiotics used, consistent with the hypothesis that membrane permeability disruption confers a non-specific pharmacokinetic advantage for intracellular drug accumulation that is not subject to the specific resistance mechanisms responsible for monotherapy regrowth.

Particularly noteworthy is the finding that D/PVA/I-1 in combination with ampicillin achieved complete sterilization of MRSA and *Klebsiella pneumoniae* ATCC BAA-2524 strains—pathogens against which ampicillin is clinically ineffective. These findings parallel results reported for other membrane-active potentiators. Quercetin in combination with colistin and amikacin demonstrated synergistic bactericidal activity (FICI 0.1875–0.5) against colistin-resistant *A. baumannii* strains, with membrane damage confirmed by nucleic acid leakage—a readout directly comparable to the crystal violet assay results presented in the current study [16]. Similarly, amorolfine demonstrated synergistic activity with colistin against *A. baumannii* (FICI = 0.094), operating through outer membrane disruption and inner membrane depolarization [17]. The convergence of these findings across structurally distinct potentiators underscores that membrane permeability disruption constitutes a mechanistically validated and reproducible strategy for overcoming resistance in both Gram-positive and Gram-negative ESKAPE pathogens [3].

The membrane permeability data obtained in the crystal violet uptake assay provided direct mechanistic evidence linking the activity of D/PVA/I-1 to disruption of cytoplasmic membrane integrity. The pronounced dose- and time-dependent increase in crystal violet uptake observed across all four tested strains is consistent with the established mechanism of action of iodine-based antimicrobial agents, which exert bactericidal effects through oxidative damage to membrane lipids and proteins [18,19]. The approximately 2.9-fold attenuation of the permeabilizing effect in the resistant *S. aureus* ATCC BAA-33591 strain relative to the susceptible *S. aureus* ATCC 6538-p, paralleling a 2-fold increase in MBC, suggests that the acquired resistance of this MRSA strain is mediated, at least in part, by mechanisms that limit membrane accessibility, specifically, altered cell wall architecture, modified teichoic acid content, or thickened peptidoglycan layers that restrict the penetration of membrane-active agents [20]. Importantly, the permeabilizing effect was not completely abolished in resistant strains but merely attenuated, providing the mechanistic basis for the residual potentiating activity observed in the checkerboard and time-kill experiments.

The differential response of Gram-positive and Gram-negative organisms in the membrane permeability assay, with crystal violet uptake reaching 170.67% in susceptible *S. aureus* versus 25.33% in susceptible *K. pneumoniae* at MBC concentrations, is consistent with structural differences between the two cell envelope types. Gram-negative bacteria possess an outer membrane that constitutes a formidable permeability barrier, restricting the access of membrane-active agents to the cytoplasmic membrane and reducing the magnitude of crystal violet uptake at equivalent drug concentrations. Control data with CTAB and polymyxin B confirmed the validity of the assay system: CTAB produced more pronounced permeabilization in *S. aureus* than in *K. pneumoniae* at equivalent concentrations (33.01% vs. 19.54% at 120 microg/mL, 120 min), whereas polymyxin B acted selectively against *K. pneumoniae* (22.98% uptake) with negligible effect on *S. aureus* (2.98%), consistent with its established LPS-binding mechanism conferring Gram-negative selectivity [21].

The totality of the data obtained suggests that the primary mechanism underlying the potentiating activity of D/PVA/I-1 is disruption of cell membrane integrity, leading to increased permeability to antibiotic molecules. This is consistent with the concept of “outer membrane permeabilizers”—one of the three principal classes of antibiotic adjuvants. Notably, the strongest synergistic interactions were observed with antibiotics with intracellular targets (chloramphenicol, tetracycline, gentamicin), rather than with cell wall inhibitors acting externally—an observation that indirectly supports a membrane-tropic mechanism of potentiation. It has previously been shown that iodine at sub-inhibitory concentrations acts on key cellular targets, including suppression of protein synthesis and disruption of enzymatic activity, which may contribute to the potentiating effect independently of direct membrane destabilization. The influence of D/PVA/I-1 on the expression of resistance genes, in particular, those encoding efflux pumps and β-lactamases, in the tested strains warrants dedicated transcriptomic analysis, as has been performed for related iodine-containing complexes [22].

The safety margin of D/PVA/I-1 can be inferred by comparing its antibacterial and cytotoxic concentration ranges. As previously reported by Turganbay et al., D/PVA/I-1 exhibited MBC values of 0.33–0.68 μg/mL against the tested Gram-positive and Gram-negative strains, meaning that the sub-inhibitory concentrations (0.5× MBC) used in synergy assays corresponded to approximately 0.17–0.34 μg/mL. In contrast, cytotoxic effects of D/PVA/I-1 toward human cells were observed only at substantially higher concentrations, in the milligram-per-milliliter range: moderate toxicity toward the MCF7 tumor cell line and PBMCs was reported at 5 mg/mL (62.4% and 52.0% cell viability, respectively), while a more pronounced cytotoxic effect on the normal MeT-5A cell line was observed at 2.5–5.0 mg/mL (13.8–23.0% cell viability) [8]. This corresponds to a difference of approximately three to four orders of magnitude between the concentrations at which antibacterial and synergistic activity occur and those required to elicit measurable cytotoxicity, suggesting a wide selectivity margin for D/PVA/I-1 and supporting its potential suitability as an antibiotic potentiator at antibacterially active, non-cytotoxic concentrations.

Taken together, the three complementary approaches employed in the present study establish a coherent mechanistic picture: D/PVA/I-1 acts as a membrane-permeabilizing adjuvant. It disrupts bacterial cytoplasmic membrane integrity in a dose- and time-dependent manner, facilitating the intracellular accumulation of co-administered antibiotics and thereby overcoming resistance mechanisms driven by reduced drug uptake, active efflux, or low intrinsic permeability. The broad-spectrum nature of this potentiation, demonstrated across nine MDR reference strains, five pathogen species, and nine antibiotic classes, distinguishes D/PVA/I-1 from class-specific adjuvants such as beta-lactamase inhibitors, which are effective only against strains harboring specific enzymatic resistance determinants [6]. The absence of antagonistic interactions across all 45 tested combinations supports the safety of incorporating D/PVA/I-1 into combination therapy regimens without risk of pharmacodynamic interference. Collectively, these properties, together with the demonstrated ability to restore clinical activity to antibiotics rendered ineffective by acquired resistance, position D/PVA/I-1 as a promising scaffold for the development of novel combination therapy strategies against MDR ESKAPE pathogen infections. Future investigations should focus on characterizing the pharmacokinetic and toxicological profile of D/PVA/I-1 in vivo, its activity in biofilm models, and its potential to suppress the emergence of secondary resistance in experimental evolution studies.

## 4. Materials and Methods

### 4.1. Materials

#### 4.1.1. Bacterial Strains

The microbial strains used in the current study were *S. aureus* ATCC 33591 (resistant strain), *A. baumannii* ATCC BAA-1790 (resistant strain), *K. pneumoniae* ATCC 700603 (resistant strain), *S. aureus* ATCC BAA-39 (resistant strain), *E. coli* ATCC BAA-196 (resistant strain), *E. coli* ATCC BAA-2523 (resistant strain), *K. pneumoniae* ATCC BAA-2524 (resistant strain), *S. pneumoniae* ATCC BAA-660 (resistant strain), *H. influenzae* ATCC 33930 (resistant strain), *S. aureus* ATCC 6538 (sensitive strain), and *K. pneumoniae* ATCC 10031 (sensitive strain).

Two clinical isolates were additionally used in this study: *Escherichia coli* SCAID WND1-2021 (resistant strain, Kazakhstan Patent No. 7713), recovered from a purulent wound swab of a patient with diabetes mellitus, and *Pseudomonas aeruginosa* SCAID PHRX1-2019 (resistant strain, Kazakhstan Patent No. 6286), isolated from the oropharyngeal mucosa of a patient with a prolonged upper respiratory tract infection. The genomes of both strains have been deposited in the NCBI database (*P. aeruginosa* SCAID PHRX1-2019 chromosome CP053686.1, *E. coli* SCAID WND1-2021 chromosome complete genome NZ_CP082831.1, plasmid complete sequence NZ_CP082832.1), and the cultures are deposited at the Research Institute for Biological Safety Problems (RIBSP, Kazakhstan).

Given their high clinical significance and frequent involvement in both hospital- and community-acquired infections, *E. coli* and *P. aeruginosa* were selected for inclusion in this study. *E. coli* is a leading cause of urinary tract, bloodstream, and wound infections, while *P. aeruginosa* is a major opportunistic pathogen associated with respiratory and healthcare-associated infections, both frequently exhibiting multidrug resistance. Accordingly, the clinical isolates *E. coli* SCAID WND1-2021 (recovered from a purulent wound swab of a diabetic patient) and *P. aeruginosa* SCAID PHRX1-2019 (isolated from the oropharyngeal mucosa of a patient with a prolonged upper respiratory tract infection) were included to reflect the clinical relevance of these species.

Bacteria were cultured to a density of 0.5 McFarland, which is equal to 1 × 108 colony-forming units per milliliter (CFU/mL). A 1:100 Mueller Hinton Broth (MHB, HiMedia Laboratories, Mumbai, Maharashtra, India) dilution was prepared to provide a final concentration of 1 × 106 CFU/mL for further assay and analysis.

#### 4.1.2. Antimicrobial Agents

Antibiotic substances Ampicillin (AMP), Cefazoline (CEF), Gentamycin (GEN), Tetracycline (TET), and Ceftriaxone (CTR) were procured from Titan Biotech. Ltd. (Bhiwadi, Rajasthan, India); Amoxicillin, (AMX) was purchased from HiMedia Laboratories (Mumbai, Maharashtra, India); Chloramphenicol (CL) and Levofloxacin (Lev) were obtained from Sigma-Aldrich (St. Louis, MO, USA); Azithromycin (AZM), Polymyxin B (PMB) and Doxycycline (DXC) were purchased from Sisco Research Lab. Pvt. Ltd. (Mumbai, Maharashtra, India). Stock solutions of antibiotics were prepared in appropriate solvent, according to solubility data at 16 mg/mL. Vehicle control containing an equivalent volume of solvent was prepared to account for solvent effects. All solutions were freshly prepared, shielded from light, and stored at 4 °C.

The dextrin/polyvinyl alcohol/iodine complex (D/PVA/I-1) was synthesized and characterized as previously described by Turganbay et al. [8].

### 4.2. Methods

#### 4.2.1. Susceptibility Testing (Method of Two-Fold Serial Dilutions)

Antimicrobial activity assay was conducted using a two-fold serial broth microdilution method according to the guidelines of the Clinical and Laboratory Standards Institute [11,23,24]. The experiment was carried out in sterile 96-well polystyrene plates (BIOLOGIX, Jinan, Shandong, China).

All wells of the 96-well plates were filled with 100 µL of Mueller–Hinton broth. Then, 100 µL of tested compound solution (antibiotic or D/PVA/I-1) was added into the first well in the row of the plate. Further serial two-fold dilutions were performed: 100 μL of the broth + sample solution from the first well (A1) was mixed and transferred into the next well (A2) in the row. Next, 100 μL of the twice-diluted solution from the second well (A2) was moved to the next well in the row (A3) and so forth, until the required number of dilutions was achieved. All experiments were performed in triplicate.

Control wells included a growth control (medium with inoculum, without test compounds) and a sterility control (medium only, without inoculum).

An aliquot of 18–24 h incubated test strain cells was harvested with a bacteriological loop, transferred to a test tube with sterile saline, and homogenized. Obtained microbial suspension density was adjusted to 0.5 McFarland, which corresponded to a cell concentration of 1.5 × 10^8^ CFU/mL (DEN-1, Latvia). To prepare working suspensions of bacteria, the stock inoculum was diluted with saline 100 times to a final concentration of approximately 1.5 × 10^6^ CFU/mL.

Thereafter, 20 μL of prepared bacterial suspensions was added into each well of the row with test items. The samples were incubated at 37 ± 1 °C for 24 h.

To determine the minimum inhibitory concentration (MIC) values, after the incubation period, 0.05 mL of a sterile 0.05% aqueous solution of resazurin (indicator) was added to each well, followed by incubation at 37 ± 1 °C for 30 min. The results of the resazurin assay were assessed colorimetrically by evaluating the viability of microbial cells under the influence of different concentrations of the tested samples. This method is based on the assessment of the metabolic activity of microorganisms, specifically on the detection (λ = 540 nm, Multiskan Ascent, Thermo Fisher Scientific, Vantaa, Finland) of the formation of pink resorufin as a result of microbial reduction of the phenoxazine dye resazurin (blue color). The MIC was recorded as the lowest concentration that prevented the color change in the medium. The experiment was carried out in triplicate [25]. The minimum bactericidal concentration (MBC) was defined as the lowest concentration of antibacterial agents that completely kill organisms. This test was performed by plating the suspension from each well of the microtiter plates into an MHA plate. After that, the plates were incubated at 37 °C for 24 h. The lowest concentration with no visible growths on the MHA plate was taken as the MBC value [26].

Antimicrobial susceptibility testing was carried out according to CLSI guidelines using the broth microdilution method. Minimum bactericidal concentrations (MBCs) were determined in triplicate with an initial inoculum of 1.5 × 10^5^ CFU/mL in saline. Following 16–20 h incubation at 37 °C, the MBC was read and defined as the lowest drug concentration that completely inhibited visible growth. To determine the bactericidal dilution, after the incubation period, 0.01–0.02 µL from each well was inoculated with a sterile loop onto pre-marked Petri dishes with Mueller–Hinton agar. Each sector on the plate corresponded to the serial number of the well containing a specific dilution. After inoculation, the Petri dishes were incubated for 18–24 h at 37 ± 1 °C.

The results were assessed based on the presence or absence of microbial growth on the surface of the solid nutrient medium. The minimum bactericidal concentration (MBC) was defined as the lowest concentration of the antimicrobial agent in the well that completely inhibited the growth of the tested microorganism on the plates.

#### 4.2.2. Synergy Testing by Checkerboard Assay

The checkerboard dilution method was performed to evaluate the inhibitory activity of combinations that were assessed based on the MIC values of the single substances. The method was adopted from a previous study [27]. Two substances’ combinations were prepared in which D/PVA/I-1 and antibiotic were twofold diluted serially in a separate 96-well plate before combining into another 96-well plate for the combination experiment. The plates were incubated at 37 °C for 18–24 h, and the MIC of the drug combination was determined.

In order to evaluate the combinatorial effect of the combined substances, the fractional inhibitory concentration index (FICI) analysis was performed to determine synergistic, additive, indifferent, or antagonistic effects [28,29]. The FICI was calculated using the following formula (1):

FICI of two-drug combination = (MIC A1)/(MIC A2) + (MIC B1)/(MIC B2),(1)

where A1 is the MIC of D/PVA/I-1 in the combination, A2 is the MIC of D/PVA/I-1 alone, B1 is the MIC of antibiotic in the combination, and B2 is the MIC of antibiotic alone. The results indicate synergism when the corresponding FICI ≤ 0.5, partial synergism when 0.5 < FICI ≤ 1, indifference when 1 < FICI ≤ 4, and antagonism when the FICI > 4 [28,29].

#### 4.2.3. Time-Kill Assay

A static time-kill assay was conducted for nine strains according to [30]. The double antimicrobial combinations D/PVA/I-1-cefazolin, D/PVA/I-1–chloramphenicol, D/PVA/I-1–gentamycin, D/PVA/I-1–ampicillin, D/PVA/I-1–amoxicillin, and D/PVA/I-1–tetracycline were tested. Bacteria (1 × 10^6^ CFU/mL) using concentrations of 0.5 MIC were inoculated in Muelle–Hinton broth containing antibiotic and D/PVA/I-1 with continuous shaking overnight at 37 °C in an atmospheric environment. One-hundred-microliter samples were drawn and then serially diluted at 0, 2, 4, 6, 8 and 10 h, and 50 µL aliquots were smeared on Mueller–Hinton agar plates. After incubating the plates overnight at 37 °C, the colonies were counted. Synergy was defined as a decrease of ≥2 log10 CFU/mL between the combination and the most efficient agent alone at 24 h. Bactericidal activity was defined as ≥3 log10 CFU/mL reduction in cell numbers compared to the initial inoculum after 24 h [12,31].

#### 4.2.4. Measurement of Permeability with Crystal Violet

The alteration in membrane permeability was also evaluated by crystal violet (CV) assay [32,33]. Bacterial cell suspensions were harvested at 4500× *g* for 5 min at 4 °C. The cells were washed twice and resuspended in 0.5 mM potassium phosphate buffer solution (pH 7.4). The washed bacterial cell suspension was incubated with Cetyltrimethylammonium bromide (CTAB) and Polymyxin B at 37 °C for 30 min. Control samples were prepared similarly without treatment. The cells were resuspended in 0.5 mM potassium phosphate buffer solution (pH 7.4) containing 10 g/mL of crystal violet after harvesting them at 9300× *g* for 5 min. After incubating at 37 °C for 10 min, the suspension was centrifuged at 13,400× *g* for 15 min, and the OD of the supernatant was measured at a wavelength of 590 nm (Tecan Sunrise, Tecan Austria GmbH, Grödig, Austria). OD of the supernatant of the normal untreated cells was used as blank. The OD value of crystal violet solution was considered as 100%. The percentage of crystal violet uptake was expressed as follows:

(OD value of sample)/(OD value of CV solution) × 100 = Crystal violet uptake (%).(2)

#### 4.2.5. Statistical Analysis

Quantitative data were analyzed using one-way analysis of variance (one-way ANOVA). Statistical processing and graphical visualization were performed using GraphPad Prism 5 software.

## 5. Patents

A utility model patent titled “Use of an iodine-containing polymer composition to enhance the antimicrobial action of antibiotics” (Patent No. 12360, Republic of Kazakhstan, published on 5 June 2026) resulted from the work reported in this manuscript.

## Figures and Tables

**Table 1:** MIC, FICI of antibiotics and D/PVA/I-1 and their combination.

Microorganism	MBC/MIC of Drug Alone (µg/mL)		Concentration of Substance (µg/mL) in Combination		FICI	Type of Interaction
			AB	D/PVA/I-1		
*S. aureus* ATCC 33591	Ceftriaxone	2000/1000 (R)	250		0.5	Synergy
	D/PVA/I-1	0.33/0.16		0.04		
	Levofloxacin	2.0/1.0 (S)	0.125		0.375	Synergy
	D/PVA/I-1	0.33/0.16		0.04		
	Gentamicin	500/250 (R)	63		0.5	Synergy
	D/PVA/I-1	0.33/0.16		0.04		
	Tetracycline	500/250 (R)	63		0.378	Synergy
	D/PVA/I-1	0.33/0.16		0.04		
	Azithromycin	2000/1000 (R)	500		0.381	Synergy
	D/PVA/I-1	0.33/0.16		0.02		
	Amoxicillin	250/125 (R)	16		0.378	Synergy
	D/PVA/I-1	0.33/0.16		0.04		
	Ampicillin	1000/500 (R)	250		0.75	Partial synergy
	D/PVA/I-1	0.33/0.16		0.04		
	Chloramphenicol	4000/2000 (R)	125		0.313	Synergy
	D/PVA/I-1	0.33/0.16		0.04		
	Doxycycline	7.8/3.9 (S)	1.95		1.0	Additivity
	D/PVA/I-1	0.33/0.16		0.08		
*S. aureus* ATCC BAA-39	Ceftriaxone	4000/2000 (R)	250		0.375	Synergy
	D/PVA/I-1	0.33/0.16		0.04		
	Levofloxacin	4.0/2.0 (S)	0.125		0.563	Partial synergy
	D/PVA/I-1	0.33/0.16		0.08		
	Gentamicin	2000/1000 (R)	250		0.5	Synergy
	D/PVA/I-1	0.33/0.16		0.04		
	Tetracycline	1000/500 (R)	63		0.376	Synergy
	D/PVA/I-1	0.33/0.16		0.04		
	Azithromycin	4000/2000 (R)	500		0.75	Partial synergy
	D/PVA/I-1	0.33/0.16		0.08		
	Amoxicillin	4000/2000 (R)	500		0.5	Synergy
	D/PVA/I-1	0.33/0.16		0.04		
	Ampicillin	125/62.5 (I)	15.63		0.5	Synergy
	D/PVA/I-1	0.33/0.16		0.04		
	Chloramphenicol	4000/2000 (R)	1000		0.75	Partial synergy
	D/PVA/I-1	0.33/0.16		0.04		
	Doxycycline	7.8/3.9 (S)	1.95		0.75	Partial synergy
	D/PVA/I-1	0.33/0.16		0.04		
*Escherichia coli* ATCC BAA-196	Ceftriaxone	1.0/0.5 (S)	0.125		0.515	Partial synergy
	D/PVA/I-1	0.68/0.34		0.09		
	Levofloxacin	2.0/1.0 (S)	0.125		0.390	Synergy
	D/PVA/I-1	0.68/0.34		0.09		
	Gentamicin	2000/1000 (R)	500		0.647	Partial synergy
	D/PVA/I-1	0.68/0.34		0.05		
	Tetracycline	128/64.0 (I)	8.0		0.390	Synergy
	D/PVA/I-1	0.68/0.34		0.09		
	Azithromycin	250/125 (I)	32.0		0.521	Partial synergy
	D/PVA/I-1	0.68/0.34		0.09		
	Amoxicillin	4000/2000 (R)	500		0.510	Partial synergy
	D/PVA/I-1	0.68/0.34		0.09		
	Ampicillin	4000/2000 (R)	1000		0.650	Partial synergy
	D/PVA/I-1	0.68/0.34		0.05		
	Chloramphenicol	500/250 (R)	64.0		0.760	Partial synergy
	D/PVA/I-1	0.68/0.34		0.17		
	Doxycycline	15.6/7.8 (S)	1.95		0.51	Partial synergy
	D/PVA/I-1	0.68/0.34		0.09		
*Klebsiella pneumoniae* ATCC BAA-2524	Ceftriaxone	1.0/0.5 (S)	0.125		0.515	Partial synergy
	D/PVA/I-1	0.68/0.34		0.09		
	Levofloxacin	1.0/0.5 (S)	0.25		0.647	Partial synergy
	D/PVA/I-1	0.68/0.34		0.05		
	Gentamicin	4000/2000 (R)	250		0.390	Synergy
	D/PVA/I-1	0.68/0.34		0.09		
	Tetracycline	16/8.0 (S)	2.0		0.515	Partial synergy
	D/PVA/I-1	0.68/0.34		0.09		
	Azithromycin	128/64.0 (I)	8.0		0.765	Partial synergy
	D/PVA/I-1	0.68/0.34		0.09		
	Amoxicillin	4000/2000 (R)	500		0.515	Partial synergy
	D/PVA/I-1	0.68/0.34		0.09		
	Ampicillin	4000/2000 (R)	1000		0.647	Partial synergy
	D/PVA/I-1	0.68/0.34		0.05		
	Chloramphenicol	4000/2000 (R)	500		0.397	Synergy
	D/PVA/I-1	0.68/0.34		0.05		
	Doxycycline	250/125.0 (I)	64		0.777	Partial synergy
	D/PVA/I-1	0.68/0.34		0.09		
*Acinetobacter baumannii* ATCC 1790	Ceftriaxone	128/64.0 (I)	8.0		0.750	Partial synergy
	D/PVA/I-1	0.33/0.16		0.04		
	Levofloxacin	64.0/32.0 (I)	4.0		0.375	Synergy
	D/PVA/I-1	0.33/0.16		0.04		
	Gentamicin	8.0/4.0 (S)	1.0		0.375	Synergy
	D/PVA/I-1	0.33/0.16		0.02		
	Tetracycline	32.0/16.0 (I)	8.0		0.750	Partial synergy
	D/PVA/I-1	0.33/0.16		0.04		
	Azithromycin	128/64.0 (I)	32.0		1.0	Additivity
	D/PVA/I-1	0.33/0.16		0.08		
	Amoxicillin	2000/1000 (R)	250		0.375	Synergy
	D/PVA/I-1	0.33/0.16		0.02		
	Ampicillin	4000/2000 (R)	500		0.500	Synergy
	D/PVA/I-1	0.33/0.16		0.04		
	Chloramphenicol	250.0/125.0 (I)	64		1.0	Additivity
	D/PVA/I-1	0.33/0.16		0.08		
	Doxycycline	16.0/8.0 (S)	4.0		0.750	Partial synergy
	D/PVA/I-1	0.33/0.16		0.04		

Note: «R»—resistant, «I»—intermediate, «S»—sensitive.

**Table 2:** Permeability of bacterial membrane in the presence of CTAB and polymyxin B, expressed as crystal violet (CV) uptake (%) = (OD of sample/OD of CV solution) × 100, where OD is the optical density measured at 590 nm.

Sample	Concentration, μg/mL	% Absorption CV		
		30 min	90 min	120 min
***Staphylococcus aureus*** **ATCC 6538-p**				
CTAB	30 (1/4 MBC)	12.88 ± 0.57	14.56 ± 0.57	16.98 ± 1.05
	60 (1/2 MBC)	17.52 ± 1.00	19.98 ± 0.57	25.65 ± 0.57
	120 (MBC)	20.01 ± 0.57	29.21 ± 0.57	33.01 ± 0.33
Polymyxin B	30 (1/4 MBC)	0.89 ± 0.02	0.99 ± 0.02	1.52 ± 0.02
	60 (1/2 MBC)	0.99 ± 0.02	1.65 ± 0.02	1.98 ± 0.02
	120 (MBC)	1.00 ± 0.02	1.50 ± 0.02	2.98 ± 0.02
***Klebsiella pneumoniae*** **ATCC 10031**				
CTAB	30 (1/4 MBC)	7.98 ± 0.57	9.20 ± 0.57	9.99 ± 1.15
	60 (1/2 MBC)	10.65 ± 0.57	12.65 ± 0.57	13.98 ± 0.57
	120 (MBC)	13.68 ± 0.57	16.54 ± 0.63	19.54 ± 0.33
Polymyxin B	30 (1/4 MBC)	8.99 ± 0.57	10.87 ± 0.57	11.05 ± 1.05
	60 (1/2 MBC)	10.55 ± 0.57	12.05 ± 1.15	12.74 ± 0.57
	120 (MBC)	14.05 ± 0.57	18.98 ± 0.57	22.98 ± 0.57

**Table 3:** Minimum bactericidal and subbactericidal concentrations of D/PVA/I-1 in relation to sensitive and multidrug-resistant test strains.

Test Culture	D/PVA/I-1 Concentration, μg/mL		
	MBC	1/2 MBC	1/4 MBC
	D/PVA/I-1		
*Staphylococcus aureus* ATCC 6538-p	0.16	0.08	0.04
*Staphylococcus aureus* ATCC BAA-33591	0.33	0.16	0.08
*Klebsiella pneumoniae* ATCC 10031	0.34	0.17	0.09
*Klebsiella pneumoniae* ATCC BAA-2524	0.68	0.34	0.17

**Table 4:** Permeability of bacterial membranes in the presence of D/PVA/I-1, expressed as crystal violet (CV) uptake (%) = (OD of sample/OD of CV solution) × 100, where OD is the optical density measured at 590 nm.

Concentration D/PVA/I-1, μg/mL	% Absorption CV		
	30 min	90 min	120 min
***Staphylococcus aureus*** **ATCC 6538-p**			
0.16 (MBC)	80.6 ± 5.51	106.67 ± 0.57	123.63 ± 2.52
0.08 (1/2 MBC)	92.5 ± 0.67	131.33 ± 1.5	151.33 ± 1.53
0.04 (1/4 MBC)	98.4 ± 2.52	144.67 ± 0.57	170.67 ± 2.31
***Staphylococcus aureus*** **ATCC BAA-33591**			
0.33 (MBC)	10.05 ± 0.58	22.33 ± 0.33	33.01 ± 0.33
0.16 (1/2 MBC)	12.65 ± 0.57	27.54 ± 1.15	47.55 ± 0.58
0.08 (1/4 MBC)	16.98 ± 0.57	23.65 ± 0.57	59.67 ± 0.57
***Klebsiella pneumoniae*** **ATCC 10031**			
0.34 (MBC)	9.20 ± 0.57	12.65 ± 0.57	13.98 ± 0.57
1.17 (1/2 MBC)	12.65 ± 0.57	16.54 ± 0.63	19.54 ± 0.33
0.09 (1/4 MBC)	13.68 ± 0.57	16.54 ± 0.63	25.33 ± 0.57
***Klebsiella pneumoniae*** **ATCC BAA-2524**			
0.68 (MBC)	9.99 ± 0.57	10.56 ± 0.57	16.98 ± 1.00
0.34 (1/2 MIC)	10.05 ± 0.58	11.67 ± 0.33	17.52 ± 0.57
0.17 (1/4 MIC)	11.20 ± 0.57	15.65 ± 0.58	20.0 ± 1.00

## Data Availability

The original contributions presented in this study are included in the article. Further inquiries can be directed to the corresponding author.
